# Chromosome remodelling by SMC/Condensin in *B. subtilis* is regulated by monomeric Soj/ParA during growth and sporulation

**DOI:** 10.1073/pnas.2204042119

**Published:** 2022-10-07

**Authors:** David M. Roberts, Anna Anchimiuk, Tomas G. Kloosterman, Heath Murray, Ling Juan Wu, Stephan Gruber, Jeff Errington

**Affiliations:** ^a^Centre for Bacterial Cell Biology, Biosciences Institute, Newcastle University, Newcastle upon Tyne, NE2 4AX, United Kingdom;; ^b^Department of Fundamental Microbiology, University of Lausanne, Bâtiment Biophore, 015 Lausanne, Switzerland

**Keywords:** chromosome segregation, ParABS, *B. subtilis*, SMC/Condensin, sporulation

## Abstract

SMC complexes, loaded at ParB-*parS* sites, are key mediators of chromosome organization in bacteria. ParA/Soj proteins interact with ParB/Spo0J in a pathway involving adenosine triphosphate (ATP)-dependent dimerization and DNA binding, facilitating chromosome segregation in bacteria. In *Bacillus subtilis*, ParA/Soj also regulates DNA replication initiation and along with ParB/Spo0J is involved in cell cycle changes during endospore formation. The first morphological stage in sporulation is the formation of an elongated chromosome structure called an axial filament. Here, we show that a major redistribution of SMC complexes drives axial filament formation in a process regulated by ParA/Soj. Furthermore, and unexpectedly, this regulation is dependent on monomeric forms of ParA/Soj that cannot bind DNA or hydrolyze ATP. These results reveal additional roles for ParA/Soj proteins in the regulation of SMC dynamics in bacteria and yet further complexity in the web of interactions involving chromosome replication, segregation and organization, controlled by ParAB and SMC.

## Significance

Accurate segregation of chromosomes is critical for cell viability. ParABS systems drive chromosome and plasmid segregation in most bacteria. The conserved SMC/Condensin complexes, loaded by ParB-*parS* in bacteria, drive chromosome arm alignment and folding. In this work we used the exceptionally high genetic tractability of *Bacillus subtilis* endospore formation to probe the role of ParA (Soj) in chromosome segregation. We demonstrate that Soj monomers are functional and act to prevent SMC/Condensin complex release onto the DNA following loading by ParB/Spo0J, at least during sporulation. Our work identifies Soj as a major regulator of SMC dynamics in bacteria, acting subsequently to DNA loading, and provides mechanistic insights into a major chromosome reorganization process that drives the initial stages of sporulation.

The stable inheritance of chromosomes is fundamental to virtually all cells. In bacteria, the lack of an overt mitotic spindle raises intriguing questions about their mechanisms of chromosome segregation and offers the possibility of targeting the process with selectively toxic antibiotics.

ParABSs are major systems involved in chromosome segregation in most bacteria and on many low-copy-number plasmids (1–3). They center on multiple *parS* sites in DNA, which are almost always located close to *oriC*, the single origin of bacterial chromosome replication (4, 5). ParB proteins (Spo0J in *Bacillus subtilis*) are DNA-binding CTP hydrolases (6–8). ParB-CTP dimers have an open configuration that, upon specific interactions with *parS*, clamp shut around DNA. Closed ParB dimers are released from *parS* sites, allowing local spreading by lateral (and possibly bridging) interactions before CTP hydrolysis and their release from DNA (6–15). The final system component is ParA (Soj in *B. subtilis*), a Walker adenosine triphosphatase (ATPase). Adenosine triphosphate (ATP) binding to Apo-ParA/Soj drives a structural change that allows its dimerization and non-specific DNA binding (16–19). Interaction between ParB/Spo0J and ParA/Soj then drives ATP hydrolysis in the latter and Soj/ParA release from DNA (17, 19, 20). The extent of ParA dimer binding on the genome varies between bacterial species. For example, in *Caulobacter crescentus*, ParA dimers bind nonspecifically over the entire nucleoid, whereas they are restricted to the *oriC* region in *B. subtilis* (21–24). One proposed model for ParAB-mediated chromosome segregation is the DNA relay system as in *C. crescentus*, which drives directed motion of one chromosome origin from the stalked to the flagellated pole (21, 25, 26). In this system, ParA dimers radiate along the length of the chromosome, with the ParB-*parS* complex being anchored to one pole. Upon DNA replication initiation, the replicated origin (ParB-*parS*) stimulates ATP hydrolysis of nearby DNA-bound ParA dimers. This releases the ParA molecules from the chromosome, which then interact with PopZ at the pole. Meanwhile, the progressing ParB/*parS* complex interacts with the next ParA dimer on the DNA, essentially allowing the *parS* to follow a retreating “cloud” of ParA on the DNA. A similar mechanism has been proposed for the segregation of ParABS plasmids (13, 27–30).

In *B. subtilis*, Soj and Spo0J are also known to have key roles in DNA replication and endospore formation in addition to chromosome segregation (22, 31, 32). For example, we have previously shown that monomer and dimer forms of Soj have opposing effects on DNA replication (inhibiting or promoting it, respectively) through direct interactions with the master initiator of DNA replication, DnaA (20, 22, 33). It then emerged that Spo0J contributes to chromosome segregation by recruiting and loading the bacterial SMC/Condensin complex (15, 34). SMC complexes align and juxtapose the left and right chromosome arms as they travel from their Spo0J-*parS* loading sites to the terminus regions, after which they are specifically unloaded by XerD (35–40). It is now recognized that ParB/Spo0J can load SMC complexes in a wide variety of bacterial species (15, 34, 41–43). In *B. subtilis*, *spo0J* mutants are also deficient in endospore formation because in the absence of Spo0J, Soj accumulates as an ATP dimer that promotes DNA overreplication (22), leading to a block in sporulation via a checkpoint mechanism involving the sporulation inhibitor, *sda* (44, 45).

**Fig. 1.**
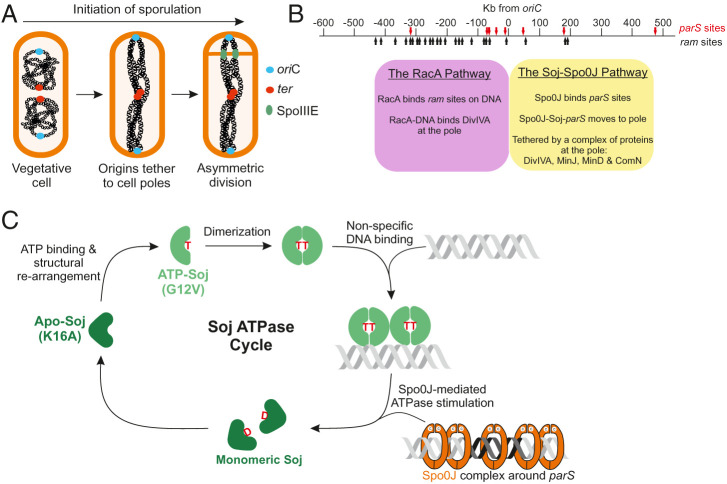
Chromosome dynamics during early sporulation. (*A*) Schematic representation of the changes to chromosome segregation during early sporulation. Chromosome origins (blue circles) become anchored to opposite cell poles while the terminus region (red circles) remain associated at midcell. This is followed by asymmetric division that defines the small prespore and large mother cell. SpoIIIE (green circles) resides in the asymmetric septum to prevent DNA scission and pump the mother cell localized portion of the bisected chromosome into the prespore. (*B*) Schematic showing the major features of the origin region required for anchoring of the chromosome to the cell poles during sporulation. There are two redundant pathways involved in capturing the origin. The first is the sporulation-specific RacA pathway, which is centered around *ram* sites (black arrows) to the left of *oriC*. The second pathway is centered around *parS* at *oriC* (red arrows). Spo0J binds *parS*, and along with Soj and a complex of polar proteins, tethers the origin region to the pole. (*C*) The Soj ATPase cycle. Upon binding to ATP, empty (Apo) Soj monomers structurally rearrange to form ATP-Soj. ATP-Soj can dimerize, which facilitates nonspecific DNA binding. Interactions between DNA-bound Soj dimers and Spo0J (bound around *parS* sites) trigger the ATPase activity of Soj, release from DNA, and a structural reversion back to the Apo form. The mutations K16A and G12V lock Soj in the Apo or ATP forms, respectively.

As well as growing vegetatively, *B. subtilis* can form endospores, with sporulation being one of the best characterized developmental systems in biology (46). The system we have used in the current study is that of sporulating *B. subtilis* since it provides a well-defined platform from which to examine the pivotal roles of Soj and Spo0J. In early sporulation, the chromosomes undergo a major reorganization to form an elongated structure termed the axial filament (or stage I) (47). A complete axial filament stretches the entire length of the cell, with origins located and anchored at cell poles and termini linked at midcell (48–51). Formation of the axial filament is critical to ensure chromosome capture upon asymmetric cell division (Fig. 1*A*) (32), which is required to enable the subsequent SpoIIIE (FtsK)-dependent segregation of the bisected chromosome into the tiny prespore (52–57). Despite its importance, precisely how the chromosome is reorganized to form the axial filament, as well as how origins are segregated to opposite cell poles, remains unclear. It is understood, however, that two redundant systems operate to anchor the segregated origins to opposite poles (32, 58). Both are dependent on DivIVA, a landmark protein that localizes to septa and cell poles (59). One of these involves the sporulation-specific RacA protein, which interacts with DivIVA and specific binding (*ram*) sites on the chromosome to the left of *oriC* (Fig. 1*B*) (32, 58, 60, 61). RacA has also been proposed to bind the chromosome and aid in its elongation across the cell (62). However, sporulation can occur via an alternate RacA-independent system, in which a number of proteins, including Soj and Spo0J, interact with DivIVA to promote capture of the *parS* region at the pole (Fig. 1*B*) (32, 63, 64). The two systems show redundancy, since only when both systems are deleted is there a near-complete abolition of polar chromosome anchoring (32, 63). Given that Soj and Spo0J are implicated in axial filament formation, and Spo0J loads SMC complexes onto the DNA, with the latter having a critical role in nucleoid organization, the ability to genetically control each step of sporulation (versus vegetative growth) offers a set of powerful tools to understand the roles of Soj-Spo0J and SMC complexes in chromosome segregation in *B. subtilis* (46).

In this work, we report a series of surprising findings that change our understanding of axial filament formation and the roles of ParAB and SMC complexes in *B. subtilis*. First, we show that ParA/Soj is active in chromosome segregation as variants, probably monomeric, that cannot bind DNA. Furthermore, we show that ParA/Soj monomers can exist in two functional states, probably corresponding to the Apo- and ATP-bound forms. The major functional difference between these forms lies in controlling SMC complex release from Spo0J-*parS* sites after loading. Finally, we show formation of the axial filament involves a major redistribution of SMC complexes along the chromosome and that the Apo- and ATP-forms of ParA/Soj control this, thus providing mechanistic insights into chromosome dynamics in bacteria.

## Results

### Wild Type Soj Protein Appears Mainly Monomeric.

**Fig. 2.**
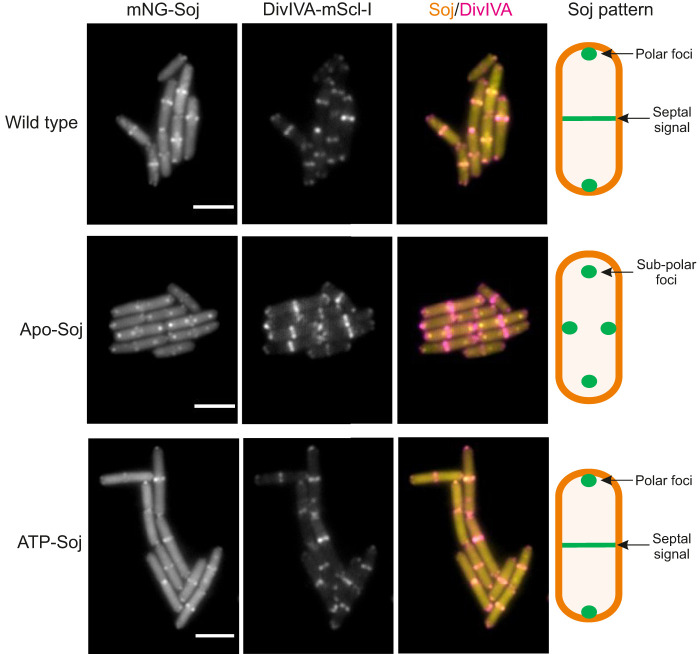
Colocalization of Soj and DivIVA. Representative images showing the localization patterns of mNG-Soj and DivIVA-mScl-I. Images were acquired 80 min after resuspension in sporulation salts. Scale bar = 3 μm. The schematic beside each set of images shows the Soj localization pattern. Strains used: DMR337, DMR339, and DMR341.

To gain insights into the function of Soj in chromosome segregation during sporulation, we prepared a fusion of Soj to the mNeonGreen (mNG) fluorescent protein (65), which is unrelated to green fluorescent protein (GFP) and is brighter than previously used GFP–Soj fusions. As shown in Fig. 2 (*Top* row, *Left* panel) early sporulating cells expressing mNG-Soj as the only functional copy of Soj showed a complex pattern with at least three detectable elements: a diffuse background signal throughout the cell; a septal signal, which appeared to be associated with recently completed vegetative septa; and an extreme polar focus, reminiscent of the localization of proteins DivIVA, MinD, and ComN, involved in the ORI-zone trapping system, or “polar complex,” described previously (63). Previous work has shown that amino acid substitutions K16A and G12V trap Soj in forms that behave as monomers in vitro and are either empty or bound to ATP, respectively (Fig. 1*C*) (20, 66). Hereafter we refer to Soj(K16A) as Apo-Soj and Soj(G12V) as ATP-Soj. As shown in Fig. 2 (*Bottom* row, *Left* panel), localization of ATP-Soj was essentially indistinguishable from the wild type fusion protein, with prominent extreme polar spots and septal bands. Apo-Soj also exhibited foci near cell poles, but they appeared less strictly polar (subpolar) than the wild type or ATP-Soj forms (Fig. 2, *Middle* row, *Left* panel). This was consistent with the notion that Apo-Soj does not interact with the putative sporulation “polar complex.”

Previous studies have suggested that both ATP-Soj and Apo-Soj are monomers in vitro (20). Given the striking similarity in localization between wild type and ATP-Soj, it was possible that these proteins may also be monomeric in vivo. To test this, we took advantage of the fact that in a *spo0J* mutant Soj accumulates in a dimeric form that binds nonspecifically to the nucleoid and not to the cell poles (22). We therefore repeated the imaging of the three mNG-Soj constructs (wild type/ATP-Soj/Apo-Soj) in the presence or absence of *spo0J*. Upon imaging of wild type Soj, foci were seen mainly at poles and septa in Spo0J^+^ cells (*SI Appendix*, Fig. S1, *Top* row, *Left* panels), as expected if the protein is mainly monomeric. To enhance the contrast between the chromosome and the cell poles, we treated the cells with nalidixic acid, which does not affect cell growth but inhibits DNA replication (67), leading to one or two chromosomes in the center of long cells. Clearly wild type mNG-Soj localized at cell poles but not significantly over the nucleoid (*SI Appendix*, Fig. S1, *Top* row, *Right* panels). Again, as expected, upon deletion of *spo0J* Soj now accumulated specifically over the isolated nucleoids (*SI Appendix*, Fig. S1, second row). Crucially, redistribution of Soj to the nucleoid did not occur in mNG-ATP-Soj or mNG-Apo-Soj strains when *spo0J* was deleted, and this was particularly clear in the + nalidixic acid panels (*SI Appendix*, Fig. S1). These results strongly support the view that the ATP-Soj and Apo-Soj variants either are monomeric in vivo or form unstable dimers that are unable to persist long enough to redistribute to the chromosome, and also that the wild type protein is normally mainly monomeric.

To test more directly for possible interactions with the sporulation polar complex (63), the strains containing the mNG-Soj fusion proteins also contained DivIVA-mScarlet-I (DivIVA-mScl-I). DivIVA is a major hub protein at the cell pole and the likely upstream target for the polar complex (63). Fig. 2 shows representative images that suggest that wild type and ATP-Soj septal bands (representing new cell poles) and extreme polar foci (older cell poles) largely colocalized with DivIVA-mScl-I. Strikingly, the Apo-Soj spots described as subpolar above indeed failed to overlay with the DivIVA-mScl-I signal. To interrogate this further, we determined the relative cellular position of Soj and DivIVA foci in every cell and then plotted these positions as fluorescence profiles in which each cell is stacked left to right by increasing cell length (*SI Appendix*, Fig. S2). This confirmed that mNG fusions of wild type and ATP-Soj colocalized tightly with DivIVA-mScl-I at midcell septa, as determined by the tight overlay of foci generating white spots in the merged profile (*SI Appendix*, Fig. S2 *A* and *C*). Apo-Soj clearly showed very little overlay with the DivIVA signal at central septa. In general, the Apo-Soj foci appeared subpolar at both outer (old) cell poles and medial (new) septa (*SI Appendix*, Fig. S2*B*). These data were further supported by quantitative analysis of the overlap in fluorescent signals between Soj and DivIVA using colocalization analysis (*SI Appendix*, Fig. S3).

Together these data support the idea that wild type Soj and the ATP-Soj variant distribute similarly in vivo, probably as monomers (22), since the localization pattern of ATP-Soj mirrored that of wild type, and was distinct from that observed when *spo0J* was deleted. Furthermore, both wild type and ATP-Soj colocalized substantially with DivIVA, especially at mid cell, consistent with the notion that Soj is recruited to the polar complex. In contrast, Apo-Soj forms subpolar foci, which generally do not colocalize with DivIVA, especially at new cell poles.

### ATP-Soj Can Support ORI-Zone Trapping during Sporulation.

**Fig. 3.**
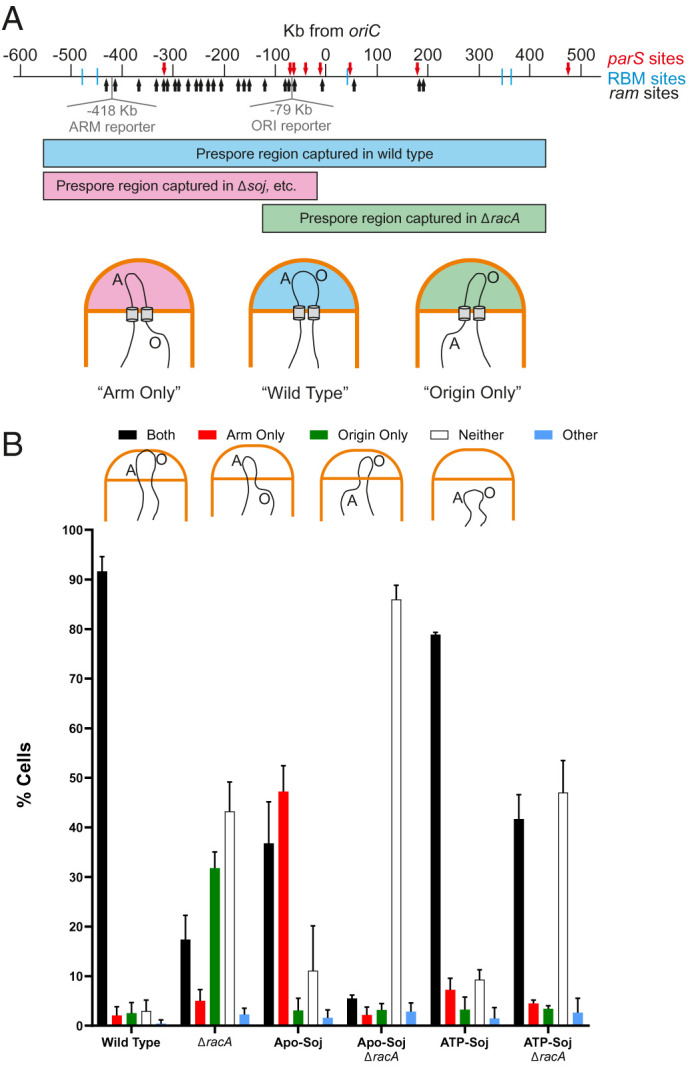
ATP-Soj and Apo-Soj have opposing effects in the chromosome trapping assay. (*A*) Schematic showing the major features of the origin region required for anchoring the chromosome to the pole. There are two redundant pathways involved in origin capture. RacA binds to *ram* sites (black arrows) in the origin proximal region, which tethers the origin to the pole. Mutations in *racA* cause an “origin only” phenotype (green). *parS* sites (red arrows) at *oriC* are the target of the Soj-Spo0J pathway that tethers the region to the pole. Mutants in *soj*, *spo0J*, or other members of the polar complex cause an “arm only” phenotype (pink). Wild type cells successfully capture both regions (blue). The genomic positions of prespore-specific reporter genes in the ORI and ARM regions are shown in gray. (*B*) Chromosome trapping assay to assess the prespore localization of the arm- or origin-localized fluorescent markers. At least 100 cells were scored per repeat (*n* = 3), and the averages are shown. Error bars show SDs. The schematic below each category panel shows the chromosome localization pattern in that condition. Strains used: DMR178, DMR179, DMR181, DMR190, DMR191, and DMR192.

Previous work has shown that a *soj* null mutant is deficient in the ORI-trapping function during sporulation (32, 63). Given that the wild type protein appears to localize similarly to ATP-Soj in vivo (Fig. 2), we wondered whether this Soj variant (or Apo-Soj) might retain ORI-trapping activity. We took advantage of an assay based on the expression of prespore-specific reporter genes placed in the “ARM” or “ORI” regions of the chromosome (34, 63) (Fig. 3*A*). When cells bearing a “frozen” mutant of the septum localized SpoIIIE translocase are induced to sporulate they form an asymmetric septum as normal. However, the bulk of the chromosome, which lies outside the prespore compartment when the polar septum forms, remains in the mother cell. Segments of DNA that are correctly located in the prespore can be detected if they contain a reporter gene dependent on the prespore-specific sigma factor, σ^F^ (34, 56, 68). The assay cells carried two reporter genes expressing fluorescent proteins, one located in the region of the chromosome dependent on RacA for efficient trapping (the ARM region; –418 Kb from *oriC*) and the other in the Soj-Spo0J-*parS*-dependent ORI region (–79 Kb from *oriC*) (Fig. 3*A*) (34, 63).

Wild type sporulating cells efficiently trapped (i.e., expressed) both ARM and ORI reporters, as expected (black bars in Fig. 3*B*), with few cells containing only one or neither reporters. Control cells bearing a *racA* deletion showed the characteristic defect (32, 58) in which less than 20% of cells correctly trapped both markers; the majority containing either only the ORI marker (“ORI-only” phenotype) or neither markers. As anticipated, cells producing Apo-Soj behaved like a *soj* null mutant (63), with an excess of ARM-only capture (red bars in Fig. 3*B*). Combining Apo-Soj with a Δ*racA* mutation resulted in an almost complete loss of trapping of either ARM or ORI markers, consistent with these mutations together abolishing both mechanisms for origin region segregation and trapping. Surprisingly, cells producing ATP-Soj were almost as proficient in trapping as wild type cells, with only a small accumulation of ARM-only or empty prespores (Fig. 3*B*). Furthermore, when combined with Δ*racA* mutant the strain was more proficient in correct trapping (black bars) than the Δ*racA* single mutant (Fig. 3*B*).

This result showed that ATP-Soj can, directly or indirectly, support movement and capturing of the origin region at the cell pole, whereas Apo-Soj cannot. Importantly, the finding that an ATP monomer form of Soj supports ORI trapping suggests that this function does not require Soj to dimerize or bind to DNA, nor does it require a functional ATPase cycle.

### Altered Chromosomal Distribution of SMC in Monomeric Mutants of *soj*.

It was notable that in the Δ*racA* background (which abolishes the redundant chromosome capture pathway) (32, 63), trapping of both the ARM and ORI markers was more frequent for the ATP-Soj variant than for wild type Soj (Fig. 3*B*) (Δ*racA* vs. ATP-Soj Δ*racA*). This suggested that despite their similar localization pattern, and although both can efficiently capture the origin region at the pole during sporulation, ATP-Soj may alter the chromosome in a way that wild type does not. Since the trapping assay is an end point readout, an increased frequency of trapping of both markers could be a result of a change in chromosome compaction/organization that results in both markers being present in the prespore upon asymmetric division. If so, it was conceivable that this could be due to an effect on chromosome condensation, perhaps involving altered loading of the SMC/Condensin complex at Spo0J-*parS* sites. To investigate this, we used chromatin immunoprecipitation coupled to deep sequencing (ChIP-Seq) to plot the distribution of SMC complexes associated with the chromosome under conditions similar to the trapping assay (i.e., T_3h_ sporulation and *spoIIIE36*). As shown in *SI Appendix*, Fig. S4, SMC complexes were detected at sites spread around the chromosome, irrespective of the form of Soj present. As expected for wild type Soj, major peaks of SMC were associated with sites roughly corresponding to the locations of the *oriC*-proximal *parS* sites (*SI Appendix*, Fig. S4 *A* and *D*), which are the major loading sites for SMC complexes. However, the two mutant strains both differed from the wild type in the extent of enrichment in the *parS-oriC* region: Substantially less enrichment of SMC was seen for the ATP-Soj form (*SI Appendix*, Fig. S4 *B* and *E*), whereas the Apo-Soj mutant had relatively more SMC over the region (*SI Appendix*, Fig. S4 *C* and *F*). Thus, the *soj* monomer mutations appear to affect SMC loading or dynamics, and again the Apo- and ATP-bound proteins had opposing effects.

### Apo-Soj Has a Dominant Negative Chromosome Segregation Defect.

**Fig. 4.**
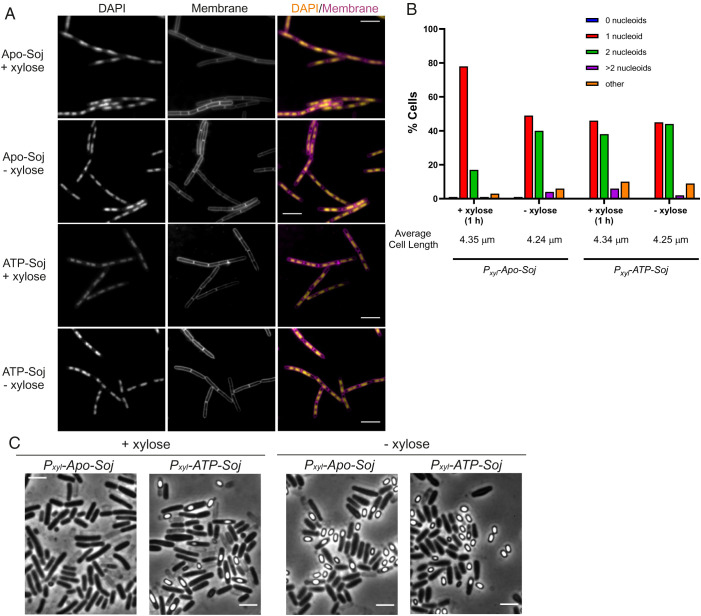
The Apo- form of Soj appears dominant negative over wild type. (*A*) Representative images of vegetative cells harboring an inducible copy of either Apo-Soj or ATP-Soj. Images were acquired 60 min after induction of the monomer mutants with 0.5% xylose. Scale bar = 5 μm. (*B*) Bar chart showing the number of completely segregated nucleoids in the Apo-Soj and ATP-Soj overexpression strains during vegetative growth. At least 150 cells were counted for each condition. (*C*) Representative images of sporulating cells containing the inducible Apo-Soj or ATP-Soj variant. Overexpression was induced by the addition of 0.5% xylose (+ xylose). −xylose = no induction. Strains used: DMR369 and DMR413.

The above observations suggested models in which Soj regulates the loading or release of SMC at Spo0J-*parS* sites and suggested that correct loading is required for ORI trapping during sporulation. If one or both proteins were trapped in forms that positively or negatively regulate SMC loading, the mutant alleles might be dominant to wild type *soj*. To test this, we engineered strains in which we could express the mutant and wild type proteins in parallel. The strain background used also carried a *dnaA(V323D)* mutation to avoid the inhibitory effects of monomeric Soj proteins (especially ATP-Soj) on the initiation of DNA replication (22, 33), as well as Δ*racA* to eliminate the alternative polar segregation pathway of sporulation. These experiments revealed, unexpectedly, a clear dominant negative phenotype for Apo-Soj but not for ATP-Soj. During vegetative growth, cells expressing Apo-Soj (i.e., +xylose) had a readily detectable alteration in nucleoid appearance, compared to cells expressing ATP-Soj or to either of the uninduced strains (i.e., –xylose) (Fig. 4*A*). In particular, induction of Apo-Soj substantially reduced the number of nucleoids per cell (Fig. 4*B*). These measurements could be influenced by changes in cell length, but the average cell length did not vary appreciably between any of the samples (Fig. 4*B*). Thus, vegetative cells expressing Apo-Soj were delayed for nucleoid separation relative to uninduced cells or cells expressing ATP-Soj.

When the same strains were induced to sporulate a much stronger phenotype was observed for Apo-Soj, in that induction with xylose virtually abolished sporulation (Fig. 4*C*). Again, no significant phenotypic effect was observed for the ATP-Soj-expressing strain. Detailed microscopic analysis of cells expressing Apo-Soj induced to sporulate revealed that they were blocked very early in the process, with a severe reduction in the formation of sporulation (polar) septa (as compared to uninduced Apo-Soj, or ATP-Soj in any condition) (*SI Appendix*, Fig. S5*A*). It is well known that perturbation of chromosome replication (e.g., by the action of dimeric Soj-based stimulation of DnaA activity) (20, 22) can block the initiation of sporulation by a “checkpoint” mechanism involving proteins Sda, KinA, and Spo0A (44, 45). Unexpectedly, however, deletion of the checkpoint gene *sda* did not suppress the sporulation defect (*SI Appendix*, Fig. S5*B*). We also asked whether the early sporulation-specific promoter of the *spoIIA* operon, which is dependent on activated Spo0A at the end of the above pathway (69), was expressed on induction of Apo-Soj, and indeed it was (*SI Appendix*, Fig. S5*C*). To our knowledge, this phenotype, in which the early sporulation operon *spoIIA* is expressed but asymmetric cell division is blocked, has been previously reported only for mutations or antibiotics inhibiting cell division directly (70–72). It seems unlikely that the Apo-Soj protein would act directly on cell division. Given that Apo-Soj expression in a *racA* mutant results in a failure to segregate and anchor the origins to the cell poles (i.e., fails to segregate the chromosome in early sporulation) (Fig. 3*B*), it seemed possible that the block in polar septation was due to incorrect positioning of the nucleoid induced by Apo-Soj, that is, some kind of nucleoid occlusion effect on cell division (73).

It was possible that the dominant negative effect mediated by Apo-Soj was due to the formation of a heterodimer between the induced Apo-Soj and the endogenous wild type copy of Soj expressed from the native locus. To test this, we repeated the dominance experiments during sporulation in strains with inducible copies of ATP- or Apo-Soj, alongside either wild type *soj*, Δ*soj*, ATP-Soj, or Apo-Soj at the native locus (*SI Appendix*, Fig. S6).

When overexpressing ATP-Soj and Apo-Soj along with wild type Soj, we saw the presence or absence of spores, respectively, as before (Fig. 4*C* and *SI Appendix*, Fig. S6). Overexpression of ATP-Soj in cells with either Δ*soj* or ATP-Soj at the native locus still resulted in the formation of spores, as indicated by the dark colony phenotype on plates (*SI Appendix*, Fig. S6*A*, +xylose plate) and the presence of phase bright spores (*SI Appendix*, Fig. S6*B*). These results further supported the notion that ATP-Soj has no dominant negative effect during sporulation.

Strikingly, when Apo-Soj was overexpressed, a sporulation defective phenotype was observed in all conditions, as indicated by the phase-pale growth on plates (*SI Appendix*, Fig. S6*A*, +xylose plate) and the absence of phase-bright spores when visualized microscopically (*SI Appendix*, Fig. S6*B*). Critically, Apo-Soj overexpression in the Δ*soj* background also abolished sporulation, demonstrating that the negative sporulation phenotype is not mediated by a heterodimer of Apo-Soj and the wild type protein. The sporulation negative Apo-Soj phenotype was also observed when the protein was expressed from the native locus in cells overexpressing ATP-Soj. Indeed, strains expressing Apo-Soj, whether from Pxyl (and + or – xylose) or from the native locus, all showed an impairment of sporulation, consistent with the view that Apo-Soj actively blocks origin movement via the ORI pathway (*SI Appendix*, Fig. S6*A*, –xylose plate and *SI Appendix*, Fig. S6*C*).

### Axial Filament Formation Is Associated with Altered SMC Distribution.

The results described above suggested a model in which Soj protein regulates the loading or sliding of SMC complexes, which then influences nucleoid configuration (i.e., formation of the axial filament) during sporulation. Correct formation of the axial filament could not only drive the origin regions toward opposite cell poles, but also configure the nucleoid to either avoid occluding the polar septum or even contribute positively to guiding septal positioning.

**Fig. 5.**
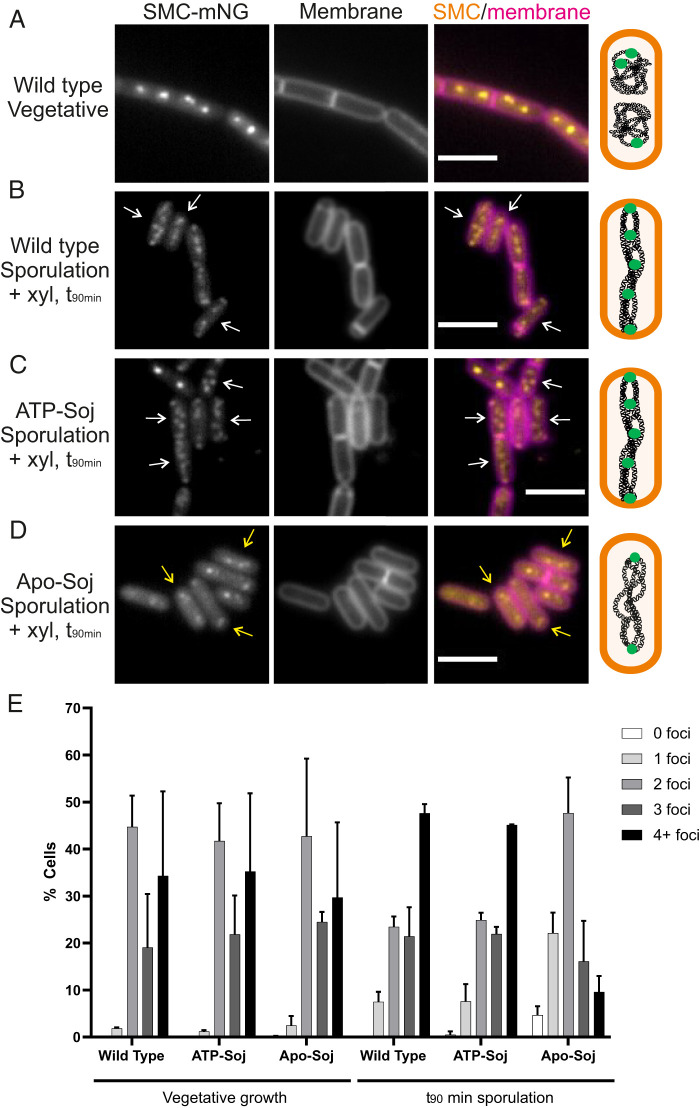
SMC distribution during sporulation. Representative images of SMC-mNG in (*A*) vegetatively growing cells with wild type *soj* or 90 min after resuspension in sporulation salts in (*B*) wild type, (*C*) ATP-soj, and (*D*) Apo-Soj mutants. In all cases, 0.5% xylose was added to cells 15 min before and throughout resuspension in sporulation salts. White arrows = cells with redistributed SMC along axial filaments; yellow arrows = cells with subpolar pairs of SMC foci. The schematic beside each set of images represents the distribution of SMC (green circles) along the chromosome in each strain. Scale bar = 3 μm. (*E*) Bar chart showing the number of SMC foci detected per cell in each mutant and time point. Between 100 and 250 cells were counted per repeat (*n* = 2), with mean scores shown. Error bars show the SDs. Strains used: DMR312, DMR314, and DMR363.

We took wild type cells bearing an SMC-mNG fusion and induced them to sporulate by the resuspension method. Samples taken immediately prior to resuspension (*t*_0_) were growing in a relatively rich medium and would be expected to have two to four copies of *oriC* (74, 75). Since SMC complexes are enriched near *oriC* due to loading at *parS* sites, two to four well-spaced foci of SMC-mNG would also be expected, and were indeed observed, in these samples (Fig. 5 *A* and *E*). Interestingly, however, after 1.5 h (*t*_90_) in sporulation medium the numbers of SMC foci increased, and in cells with multiple foci they were distributed across the full length of the cell (Fig. 5 *B* and *E*). These time points correspond to the period when cells are progressing through the early stages of sporulation. During this process, ongoing rounds of replication are completed, generating cells with two complete chromosomes (75). Meanwhile, the axial filament structure forms, in which the two *oriC* regions move to the extreme poles of the cell, while the termini remain together close to midcell. These observations are consistent with previous work showing that ScpB-YFP localizes along the chromosome arms during early sporulation (39). They also raised the exciting possibility that redistribution of SMC along the chromosome contributes to axial filament formation and that Soj might play a role in regulating this process.

### Apo-Soj Interferes with the Redistribution of SMC Protein during the Early Stages of Sporulation.

In parallel with the above experiment, we also examined the effects of ectopic expression of Apo- and ATP-Soj on SMC localization (Fig. 5). Expression of ATP-Soj had little effect on the results, and both SMC focus numbers and distribution were similar to those of the wild type (Fig. 5 *C* and *E*). However, xylose induction of Apo-Soj gave a strikingly different result: At *t*_90_ most cells still had only two widely spaced foci, presumably corresponding to the two copies of *oriC* (Fig. 5 *D* and *E*), suggesting that Apo-Soj interferes with the redistribution of SMC complexes associated with axial filament formation. To test whether RacA protein or a native copy of Soj was needed for this effect, we repeated the experiments in strains deleted for the corresponding genes. As shown in *SI Appendix*, Fig. S7, neither of these mutations affected the number of spots per cell, so it appears that Apo-Soj itself actively prevents the redistribution of SMC.

Although cells with the Apo-Soj mutation at the native locus (i.e., not overexpressed and no wild type protein) are not blocked for septum formation, we wondered whether they might nevertheless show a perturbation in localization of SMC. As shown in *SI Appendix*, Fig. S8, these Apo-Soj mutant cells also showed a reduction in numbers of SMC foci during the early stages of sporulation, whereas cells with the ATP-Soj mutation resembled the wild type control. These findings suggested that the Apo-Soj and ATP-Soj mutant proteins have contrasting effects on the loading or release of SMC complexes from the origin region during sporulation.

### SMC Complexes Are Differentially Enriched at *parS* and along the Chromosome Arms by Apo-Soj.

**Fig. 6.**
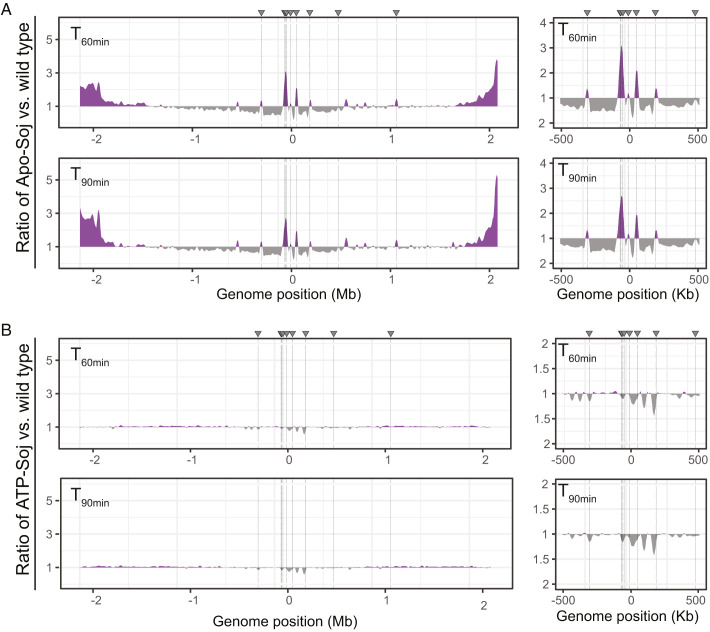
SMC complexes are differentially enriched at *parS*. ChIP-Seq was conducted against ScpB. Ratio plots show the relative enrichment (compared to wild type) of (*A*) Apo-Soj and (*B*) ATP-Soj. For each bin, normalized read counts for Apo-Soj or ATP-Soj were compared to those of the wild type for that position. If the mutant/wild type ratio was >1, values were plotted above the genome axis (purple areas). If the mutant/wild type ratio was <1, the inverse ratio was plotted below the genome axis (gray areas). T = time (min) after resuspension in sporulation salts. Gray arrows and dashed lines indicate the eight *oriC parS* sites. Panels to the right show a close-up of the *oriC* region (±500 Kb). Strains used: DMR312, DMR314, and DMR363.

As well as monitoring the effects of ectopic expression of Apo- and ATP-Soj on the cellular localization of SMC by microscopy during axial filament formation (i.e., *t*_90_) (Fig. 5), we conducted whole genome ChIP-Seq at these time points to establish the distribution of SMC across the entire genome in these cells (Fig. 6 and *SI Appendix*, Fig. S9).

*SI Appendix*, Fig. S9 shows the whole genome plots for each strain and condition. Interestingly, there were several peaks (green arrows) that appeared specifically after 60 and 90 min of sporulation, irrespective of whether the *soj* monomers were induced, suggesting that these occur when the chromosome reorganizes into the axial filament during sporulation. Based on the observation of specific peaks during sporulation, we analyzed the genomic context of all SMC enrichment peaks that were >4× during sporulation (*t*_60_ and *t*_90_) as compared to *t*_0 min_ (Table 1). The strongest enrichments were consistent in all conditions and corresponded mainly to genes whose products are typically membrane integrated/associated and that are highly transcribed in the first few hours of sporulation. In principle, these could represent roadblocks or pause sites for loop extrusion by SMC complexes.

**Table 1.** SMC is enriched along the genome during sporulation compared to vegetative growth

Gene/operon	Condition enriched	Function	Cellular location
*resA-E*	WT ± xyl ATP-soj ± xyl	*resA*: biosynthesis of cytochrome *c* *resB-C*: heme transporters *resD-E*: two-component regulator of metabolism (aerobic vs. anaerobic)	M
*dppA-E*	All	*dppA*: peptidase (cell wall metabolism) *dppB-E*: ABC transporters for dipeptides	M/E
*kinE*	All	Kinase involved in initiation of sporulation (two-component sensor)	M
*ctaC-G*	All	*ctaC*: subunit II of cytochrome *c* oxidase *ctaD*: subunit I of cytochrome *c* oxidase *ctaE*: subunit III of cytochrome *c* oxidase *ctaF*: subunit IV of cytochrome *c* oxidase *ctaG*: essential to form a functional cytochrome *c* oxidase	M

Table shows genes/operons at which SMC complexes were enriched more than fourfold during sporulation as compared to vegetative growth (_t0 min_) based on ChIP-Seq against ScpB.

M = membrane localized; E = extracellular/secreted.

All information on gene function was adapted from Subtiwiki (subtiwiki.uni-goettingen.de/).

Notably, the induced Apo-Soj ChIP-Seq profiles (*SI Appendix*, Fig. S9*C*, +xylose, *t*_60 min_ and *t*_90 min_) showed no clear gradient of SMC enrichment along the chromosome arms (outside the *oriC* peaks), suggesting that SMC distribution around the chromosome is altered in the presence of Apo-Soj. However, the presence of peaks of SMC enrichment (*SI Appendix*, Fig. S9*C* and Table 1) did suggest that some SMC complexes are present along the arms, although these were not evident as detectable SMC foci (Fig. 5). Furthermore, because these peaks were seen in all conditions, they probably represent general roadblocks or sites of enrichment for SMC as the chromosome reorganizes into an axial filament at the onset of sporulation.

Upon analysis of the distribution of SMC in Apo-Soj as a ratio compared to the wild type, several striking features emerged. First, there was a strong enrichment of SMC around *parS* sites in the Apo-Soj mutant (Fig. 6*A*). Despite this increase, there was generally less SMC on the chromosome arms in the first ∼1 Mb moving away from the *oriC*, suggesting that SMC complexes already on the chromosome before sporulation (and induction of Apo-Soj) continue to translocate, while newly loaded complexes become trapped at *parS* sites, leading to a gradual decrease (from *ori*- toward -*ter*) along the arms. Changes in SMC distribution were also noticeable with ATP-Soj (Fig. 6*B*). Here, it appeared that there was generally less SMC around the *parS* sites but a largely similar distribution of SMC along the chromosome arms at both time points during axial filament formation (Fig. 6*B*).

A similar pattern was detected when ChIP-qPCR was conducted against two independent *parS* sites (*SI Appendix*, Fig. S10). The qPCR also confirmed that there is no clear enrichment of SMC at the terminus in the *soj* mutants (*SI Appendix*, Fig. S10).

Taken together, these data suggest that SMC complexes are recruited to *parS* sites in the Apo-Soj mutant, but efficient translocation away (i.e., release) from the *oriC* region is blocked, ultimately leading to a depletion of SMC along the chromosome arms and a loss of the normal SMC gradients from *oriC* to *ter*. This further supports the notion that a loss of SMC along the chromosome arms is associated with an inhibition of origin segregation to the pole, which fails in Apo-Soj mutants. This was not the case for the ATP-Soj mutant, in which SMC complexes were depleted around *parS*, suggesting that translocation away from *oriC* is permitted, and may be promoted or increased in this mutant, leading to segregation of *oriC* regions toward the cell poles. Furthermore, these data establish that monomeric Soj proteins can act as regulators of SMC release in bacteria, which act after SMC/Condensin loading at *parS* by Spo0J.

### Apo-Soj Inhibition of ORI Trapping Is Dependent on Interaction with Spo0J.

Soj and the SMC complex are both known to interact with the N-terminal region of Spo0J (15, 19). Gruber and Errington (15) previously identified a mutation, *spo0J(L5H)*, which abolishes the stimulation of Soj ATP hydrolysis, presumably by preventing the N-terminal unstructured region of Spo0J from interacting with the active site of the Soj dimer. Since neither ATP-Soj nor Apo-Soj can bind DNA (17, 20), the apparent regulation of SMC redistribution by these Soj variants could conceivably operate through an interaction with the N-terminus of Spo0J. If so, the *spo0J(L5H)* mutation might interfere with that regulation. To test this, we returned to the ORI trapping assay with the *soj* and *spo0J* mutants expressed from their native loci. As shown in *SI Appendix*, Fig. S11, the *spo0J(L5H)* mutation substantially increased the trapping of both ORI and ARM markers for Apo-Soj. Once again, ATP-Soj had the opposite effect on trapping in the *spo0J(L5H)* background, albeit mildly. Taken together, these data suggest that both Apo-Soj and ATP-Soj interact with the N-terminus of Spo0J and that this interaction is critical for the effects of these mutant proteins on chromosome capture.

## Discussion

### Origin Segregation in sporulating *B. subtilis* Does Not Require the Dimeric Form of Soj/ParA.

The *soj-spo0J* locus of *B. subtilis* has long been recognized to play a role in chromosome segregation (31, 64), but its detailed functioning has been difficult to unravel, in part because of the complex phenotypic effects of mutations on chromosome replication and sporulation. Most bacteria are now known to have a *parABS* (*soj-spo0J*) system, almost always located close to *oriC* (5). The system of *C. crescentus* is probably the best understood mechanistically, as described at the beginning of this article. Our results clearly show that the *B. subtilis* system deviates from the *C. crescentus* paradigm, at least during sporulation. In particular, wild type *B. subtilis* Soj/ParA appears to reside in a form that closely resembles ATP-Soj and is probably monomeric (Figs. 1 and 2) (22). Since Spo0J is the activator of Soj-dimer ATPase activity, mutants that lack *spo0J* lead to wild type Soj accumulating as a dimer and binding to the nucleoid (*SI Appendix*, Fig. S1), in a manner that is indistinguishable from that of the ATP-locked Soj-dimer mutant (16, 22). Strikingly, neither ATP-Soj nor Apo-Soj binds over the nucleoid in a Δ*spo0J* mutant (*SI Appendix*, Fig. S1). These data, combined with the established in vitro data (20), support the view that the ATP-Soj and Apo-Soj proteins are likely to be monomeric in vivo, or at least fail to form stable dimers that persist and bind to the DNA.

The chromosome segregation defect in *Δsoj-spo0J* mutants is relatively mild and evident mainly during sporulation when the partially redundant RacA system is abolished (Fig. 3) (32, 63). Nevertheless, Soj and Spo0J appear to have all the biochemical properties needed for plasmid/*Caulobacter*-like segregation function, including the ATP-dependent dimerization and DNA binding properties of Soj. Furthermore, the *soj-spo0J* locus is sufficient to stabilize an otherwise unstable plasmid in *E. coli* (16, 76). However, our results have now shown that the prominent extreme movement of *B. subtilis* origins toward the cell poles during sporulation does not require dimeric, DNA-bound Soj. Why then is the dimerization and nonspecific DNA binding retained? As well as a regulated switch in controlling DNA replication initiation (20, 22), earlier work has shown that cells with *soj* and/or *spo0J* mutations are affected in an early step in origin separation in vegetative cells, which might account for the synthetic lethal effect observed when mutations in either gene were combined with an *smc* deletion (77). Thus, it is possible that a *Caulobacter*-like DNA relay system operates to drive the initial separation of origins immediately after the initiation of replication in vegetative cells. Nevertheless, our results suggest that the sporulation system is functionally quite different from that of the plasmid and *Caulobacter* systems.

### Apo- and ATP-Bound Monomers of Soj Have Differentiated Functions.

Virtually all previous work on Soj/ParA proteins has focused on their activity in the dimeric, DNA-bound state (1). However, we previously reported that an ATP-bound but monomeric form of Soj (i.e., ATP-Soj) has potent inhibitory activity against DnaA (22). This activity is now well established as a direct protein–protein interaction by both biochemical and structural studies (20, 33). The Apo-Soj mutant has a much weaker, barely detectable inhibitory activity against DnaA (20), which suggested that this form of Soj might be inactive. However, our results presented here categorically establish that Apo-Soj is an active form of the protein: It strongly inhibited sporulation, as well as generating a clear delay in nucleoid separation in vegetative cells. ATP-Soj does not have this activity. It therefore appears that ATP-Soj and Apo-Soj are not inactive forms, and in fact they have clearly differentiated and potent activities. Structural studies of various Soj/ParA proteins have revealed two distinct conformational states (17, 19), and it seems possible that the ATP-Soj and Apo-Soj proteins may mimic these alternative states. Furthermore, our data suggest that the cellular functioning of wild type Soj will involve regulated switching between these states.

### Involvement of SMC/Condensin in Axial Filament Formation and Its Regulation by Soj/ParA.

Formation of the axial filament has long been recognized as the first detectable morphological change following the onset of endospore formation in *Bacillus* (47, 78, 79), but the mechanisms underlying this process have proven difficult to dissect. A key early finding was that *oriC* regions of the chromosome migrate toward the extreme cell poles as the axial filament forms (48). This led to models in which the migration of origins toward opposite cell poles was responsible for the elongation of the chromosomes (80). Several factors involved in migration of the *oriC* region to the pole have been identified, particularly RacA (32, 58) and a series of proteins that appear to work via an interaction with the *soj-spo0J-parS* system (32, 63, 81). Until now we had assumed that the latter system would work by a kind of DNA relay mechanism involving dimeric DNA-bound Soj and the ATPase stimulating activity of Spo0J. However, our results show that Soj does not need to dimerize to support origin movement, and we have identified a change in the distribution of SMC, regulated at least in part by Soj, as the likely driving force for axial filament formation and origin migration toward the cell poles (at least in the absence of RacA, see below).

The change in distribution of SMC during sporulation is evident as an increase in the number of foci, which become distributed along the length of the cell (Fig. 5) (39). It is well established that in the axial filament there are two complete chromosomes, with origin regions at each pole and the termini maintained close together at about midcell (32, 48, 50, 75, 78, 82). Origin-associated SMC foci are thought to occur because this is where they are loaded (15, 34). SMC complexes then migrate away toward the terminus, meanwhile aligning the left and right chromosome arms (37–39). The intermediate foci seen in the axial filament might represent arm localized “pause sites,” which correlate with sporulation-specific peaks of SMC enrichment observed by ChIP-Seq. These peaks occurred at the locations of genes encoding transmembrane or secreted proteins and protein complexes that are highly transcribed during sporulation. Coupled transcription–translation and membrane insertion could conceivably generate roadblocks that arrest or delay SMC loop extrusion. It will be interesting to explore whether these accumulations contribute to the changes in chromosomal topology associated with axial filament formation. Importantly, failure of SMC to achieve this sporulation-specific chromosomal redistribution upon Apo-Soj expression was associated with a block in polar septation, suggesting that chromosome dynamics contribute to the switch in positioning of the division septum during sporulation. As a *soj-spo0J* double mutant is able to form spores (31, 32), it was possible that RacA may support loading of SMC in a Spo0J-independent manner. However, it has previously been shown that in the absence of *soj-spo0J*, SMC (as monitored by a ScpB-YFP fusion) fails to localize over the chromosome during early sporulation (39), suggesting that RacA alone (which is present in these cells) is not able to facilitate SMC redistribution. SMC foci have also been observed along the length of fully replicated *C. crescentus* chromosomes, which also radiate across the cell and have origins anchored at opposite cell poles (83). It may be that distribution of SMC along the chromosome is crucial for forming an elongated nucleoid structure that traverses the entire cell axis.

It is well established that SMC complexes are loaded onto the chromosome at the origin region by Spo0J bound around *parS* sites in many bacterial species (3, 15, 34, 41, 42). It has recently been proposed that loading results from a direct interaction between the SMC joint and the N-terminal CTP-binding domain of Spo0J (84). At some point, SMC complexes will become released from the loading site and translocate along the chromosome to facilitate chromosome segregation and organization, as previously mentioned (37, 39). Our data suggest that Apo-Soj plays an active role in regulating this process, in which it prevents the release of SMC complexes from Spo0J-*parS*. In turn, this leads to an increase in the amount of SMC trapped around Spo0J-*parS* (relative to wild type) (Fig. 6*A*) and a concomitant reduction in movement of the ORI region to the cell pole during sporulation (Fig. 3*B*). It remains unclear whether ATP-Soj promotes SMC release from around Spo0J-*parS* or plays no active role as a positive or negative regulator and instead is solely involved in DNA replication regulation (22, 33).

More work will be needed to test precisely how Soj protein regulates SMC loading or release into sliding mode. Nevertheless, our results appear to have identified yet another link in the complex web of interactions involving Soj, Spo0J, and SMC/Condensin, which connect chromosome replication, segregation, and sporulation in *B. subtilis*. They also identify roles for both Apo- and ATP-bound forms of monomeric Soj, and provide mechanistic insights into axial filament formation and spore septum positioning.

## Materials and Methods

### Bacterial Strains and Growth Conditions.

All experiments in this study used the model gram-positive bacterium *B. subtilis*, with the genetic backgrounds 168CA and 168ED.

A full list of strains, plasmids, and oligonucleotides generated or used in this study are listed in *SI Appendix*, Tables S1–S3.

In general, strains were grown on nutrient agar (Oxoid) for growth on plates or in Luria–Bertani or casein hydrolysate (CH) liquid medium. *B. subtilis* strains were transformed by inducing competency as described by Hamoen et al. (85). Supplements included (when required) 0.1–1% xylose,1 mM isopropylthio-β-galactoside, and 20 μg/mL tryptophan. Antibiotics were added to solid and liquid medium, when required, at the following final concentrations: 0.5–1 µg/mL erythromycin, 12.5–25 µg/mL lincomycin, 100 µg/mL ampicillin, 5 µg/mL chloramphenicol, 50 µg/mL spectinomycin, 2–5 µg/mL kanamycin, 6–10 µg/mL tetracycline, and 10 µg/mL zeocin.

### Sporulation of *B. subtilis.*

Strains of *B. subtilis* were induced to sporulate via the resuspension method (86–88). In summary, cultures (3–10 mL) were inoculated from fresh plates and grown overnight at 30 °C in CH medium containing the appropriate antibiotic. The following day, cultures were diluted to an optical density (OD_600_) of 0.1 in fresh CH medium and grown at 30 °C or 37 °C until the OD_600_ reached 0.8. Cultures were then pelleted and resuspended into an equal volume of A+B sporulation salts, which marked *t*_0_ of sporulation. All subsequent time points for sporulation (in min or h) are measured similarly (e.g., *t*_90 min_ marks 90 min after resuspension in sporulation salts).

### Microscopy.

In all microscopy experiments, cells were imaged with a Nikon Ti microscope equipped with a Nikon CFI Plan Apo DM Lambda 100× oil objective; MetaMorph v7.7 software (Molecular Devices); Photometrics Prime or BSI sCMOS cameras; and a Sutter Instruments Lambda LS xenon arc or CoolLED pE-300/pE-4000 LED light sources. Images were processed and analyzed in FIJI (https://imagej.net/Fiji) (89). Details of image analysis can be found in the *SI Appendix*.

For single time point imaging, overnight cultures of vegetatively growing cells were diluted to OD_600_ = 0.05–0.1 and grown for at least two cell doublings before imaging. For sporulation experiments, cells were imaged at appropriate times following resuspension into sporulation salts (see figures for details). In all cases, 0.5-µL cells were mounted onto microscope slides containing a thin layer of 1% agarose in water (supplemented with 10% medium, where appropriate) and covered with 0.13–0.17 mm coverslips (VWR). Nucleoids were visualized with 1 µg/mL DAPI (Sigma Aldrich), and membranes were visualized by adding 0.5 µg/mL fn5-95 (Molecular Probes) to the agarose pad or cells. To minimize the nonspecific binding of fn5-95, coverslips were precoated with a large droplet of polydopamine (Sigma Aldrich) (2 mg/mL dopamine in 5 mM Tris⋅HCl, pH 8.5) before sequential washing (twice) in deionized water (90). The chromosome trapping assay was conducted as described previously (63) (see also *SI Appendix*).

### Nalidixic Acid Experiment.

Strains from overnight cultures were diluted to an OD_600_ of 0.05 in fresh Luria–Bertani medium and grown at 37 °C until OD_600_ reached 0.6. Cells were then diluted back to an OD_600_ of 0.2 in the presence of nalidixic acid (10 μg/mL final) and grown for 50 mins before visualization by fluorescence microscopy. Chromosomes were labeled with DAPI (1 μg/mL final).

### Chromatin Immunoprecipitation Coupled with Deep sequencing (ChIP-Seq).

For the single-time-point ChIP-Seq, 10-mL overnight cultures of strains DMR178, DMR179, and DMR181 grown in CH media were diluted to OD_600_ = 0.1 in 50 mL fresh CH. Each strain was then grown and induced to sporulate by the resuspension method. After 3 h, each sample was cross-linked with formaldehyde (1% final) at room temperature for 30 min with shaking every 10 min. Cross-linked pellets were harvested by centrifugation and washed twice in phosphate-buffered saline before rapid freezing in liquid nitrogen and storage at −80 °C until further processing

For the time-course ChIP-Seq, 10-mL overnight cultures of strains DMR312, DMR314, and DMR363 grown in CH media were diluted to OD_600_ = 0.1 in 100 mL fresh CH. Strains were then induced to sporulate by the resuspension method. Upon resuspension in A+B sporulation salts, each sample was split into 2× 50 mL samples, with expression of the ectopic copy of *soj* being induced in one of each pair by the addition of 0.5% (final) xylose. Each sample was handled independently throughout. At *t*_0 min_, t_60 min_ and *t*_90 min_ of sporulation, 15 mL was removed from each culture and cross-linked with formaldehyde (1% final) for 30 min at room temperature with shaking every 10 min. Each sample pellet was washed twice in phosphate-buffered saline, before rapid freezing and storage at −80 °C until further processing.

Immunoprecipitation and sequencing were conducted as described previously (91); for method details, please see *SI Appendix*. Raw data can be accessed via the NCBI Gene Expression Omnibus (92).

### Quantification and Statistical Analysis.

For all experiments we had at least two biological replicates. The number of analyzed cells (*N*) for each particular experiment or repeat are indicated in each figure. Means were generated based on repeats, and error bars are shown as SDs.

## Supplementary Material

Supplementary File

## Data Availability

ChIP-Seq data have been deposited in NCBI GEO [GSE192868 (92)].
