# Supplementary material for: Chromosome remodelling by SMC/Condensin in B. subtilis is regulated by monomeric Soj/ParA during growth and sporulation
Source: Proc Natl Acad Sci U S A. 2022 Oct 7;119(41):e2204042119. doi: 10.1073/pnas.2204042119 (PMC9564211; doi:10.1073/pnas.2204042119)
Supplement: Supplementary File [file pnas.2204042119.sapp.pdf]

Supporting Information for

Chromosome remodelling by SMC/Condensin in *B. subtilis* is regulated by monomeric Soj/ParA during growth and sporulation

David M. Roberts, Anna Anchimiuk, Tomas G. Kloosterman, Heath Murray, Ling Juan Wu, Stephan Gruber, Jeff Errington

Corresponding Author: Jeff Errington. Email: Jeff.Errington@newcastle.ac.uk

This document file includes:

SI Materials and Methods

SI Figures 1-11

SI Tables 1-3

SI Materials and Methods

Chromosome Trapping Assay during Sporulation

Three hours after the induction of sporulation by the resuspension method, cells were analysed by microscopy. Prespores, identified by membrane staining with FM5-95, were scored for the expression of YFP and/or CFP and categorised as expressing both markers, YFP only, CFP only, neither marker, or as other (denoting any other pattern e.g. expression of markers in the wrong/both compartments). The presence of a specific point mutation in SpoIIIE (*spoIIIE36*) prevented the translocation of the bisected prespore chromosome from its initial capture orientation, allowing the expression and maturation of the prespore-specific fluorescent markers.

Image Analysis

In all cases, FIJI software (https://imagej.net/Fiji) (NIH) was used to process and analyse imaging data (1).

SMC-mNG spot detection

Individual SMC-mNG foci were assigned as "spots" in a semi-automated, non-biased manner by using the TrackMate plugin in Fiji (2). In TrackMate, the Laplacian of Gaussian filter was applied, and an estimated blob diameter of 5 pixels and a threshold of 250 grey levels was used to assign all SMC-mNG spots in a field of view. Following background subtraction and generation of a composite image merging the green and red (i.e., SMC-mNG and membrane) channels, the assignment of individual spots on the merge image was generated. The channel representing the SMC-mNG foci was selected as the active channel for spot detection in the software. The generated image was then saved, and the detected spots/cell counted manually.

Fluorescence Profile generation for mNG-Soj and DivIVA-mScl-I

Images of mNG-Soj (WT, Apo- and ATP variants) and DivIVA-mScl-I were loaded into FIJI. Foci of Soj and DivIVA were detected as spots using the TrackMate plugin with the Laplacian of Gaussian filter. An estimated blob diameter of 7.5 and 8.0 pixels was used for Soj and DivIVA images, respectively, and a background threshold of 750 grey levels was applied to all images. Spots were then exported as ImageJ ROIs, which generated an image containing just the assigned spots. A mask was applied prior to image smoothing. This process was then repeated for every image, position and both Soj and DivIVA channels for every strain and time point. This removed all non-specific signal apart from the software-assigned spots that represent the position of Soj/DivIVA foci.

To generate fluorescence profiles, all phase images and the assigned Soj and DivIVA spots for each imaging field were merged into an hyperstack for each strain, generating a stack each for wild type, ATP-Soj and Apo-Soj. The images were analysed, and profiles automatically assigned to each cell using the Coli-Inspector tool (version 04d) in the ObjectJ plugin for Fiji (3). In brief, each hyperstack of images were individually selected as the linked image in the ObjectJ Coli-Inspector tool, and then filaments were automatically marked using the software. Following the generation of sorted and qualified maps, the corresponding profiles were saved and processed manually as greyscale or pseudocoloured images.

ChIP-Seq

Each sample pellet (see Methods and Figures for details) was resuspended in 2 ml cold PBS and adjusted to 4 OD_{600} units (4 ml at OD_{600}=1). Adjusted sample pellets were resuspended in TSEMS buffer (50 mM Tris pH 7.4, 50 mM NaCl, 10 mM EDTA pH 8.0, 0.5 M sucrose and PIC (Sigma)) supplemented with 10 mg/ml lysozyme from chicken egg white (Sigma) and incubated for 30 min at 37°C with vigorous shaking. Resulting protoplasts were collected by centrifugation, washed twice with TSEMS, resuspended in 1 ml TSEMS and split into 3 aliquots. Pelleted samples were rapidly frozen in liquid nitrogen for storage at -80°C until further processing.

For immunoprecipitation (IP), the sample pellets were resuspended in 2 ml buffer L (50 mM HEPES-KOH pH 7.5, 140 mM NaCl, 1 mM EDTA pH 8.0, 1 % (v/v) Triton X-100, 0.1 % (w/v) Na-deoxycholate, 0.1 mg/ml RNaseA and PIC (Sigma)) and transferred to 50 ml round bottom tubes. Sonication was performed using a Bandelin Sonoplus with an MS72 tip (90 % pulse and 35 % power output) for 3 rounds of 20 sec. Lysates were transferred to 2 ml tubes and, after centrifugation for 10 min at 21,000 g at 4°C, 800 µl of the supernatant was collected for IP and 200 µl was kept as whole-cell extract (WCE) at -20°C.

Prior to IP, the anti-scpB antibody serum (Gruber Lab) was incubated with Protein G coupled Dynabeads (Invitrogen) in a 1:1 ratio for 2.5-3.5 hrs at 4°C with rotation. The antibody-bead mixture was then washed and resuspended in buffer L, prior to adding 50 µl to each IP sample, which were incubated for 2.5-3 hrs at 4°C with rotation. Next, the samples were washed with the following buffers: buffer L, buffer L5 (buffer L containing 500 mM NaCl), buffer W (10 mM Tris-HCl pH 8.0, 250 mM LiCl, 0.5 % (v/v) NP-40, 0.5 % (w/v) Na-deoxycholate, 1 mM EDTA pH 8.0), and buffer TE (10 mM Tris-HCl pH 8.0, 1 mM EDTA pH 8.0). Finally, for crosslink reversal, the IP samples were resuspended in 520 µl buffer TES (50 mM Tris-HCl pH 8.0, 10 mM EDTA pH 8.0, 1 % (w/v) SDS) and transferred to 1.5 ml screw-cap tubes. WCE samples were thawed and 300 µl of TES and 20 µl of 10 % SDS were added. Both tubes were incubated overnight at 65°C with vigorous shaking.

To purify the DNA, 500 µl phenol:chloroform:isoamyl alcohol mix (Sigma) was added to the IP and WCE tubes, mixed vigorously and centrifuged for 10 min at 13,000 rpm (at room temperature). 450 µl of aqueous phase was collected and precipitated with 1 ml 100 % ethanol in the presence of 45 µl NaOAc (Sigma) and 1.2 µl of Glycoblue (Invitrogen) for 20 min at -20°C. DNA pellets were collected by centrifugation and resuspended in 100 µl EB (Qiagen) by vigorous shaking for 10 min at 55°C. Final purification was performed using a PCR purification kit (Qiagen), eluting the DNA in 50 µl EB. Prior to deep sequencing, the success of the IP was verified with qPCR.

For deep sequencing, the DNA libraries were prepared by the Genomic Facility at CIG, UNIL, Lausanne. Briefly, the DNA was fragmented by sonication (Covaris S2) until the DNA was sheared to 220-250 bp. The Ovation Ultralow Library Systems V2 kit (NuGEN) including 15 cycles of PCR amplification was used to prepare the DNA libraries. 12-15 million sequence reads per sample were obtained on a HiSeq4000 (Illumina) with 150 bp read length.

<u>ChIP-Seq read profiling</u>

The *B. subtilis* genome NC_000964.3 was used to map the reads using bowtie2 (--very sensitive-local mode). Downstream data analysis was conducted using SeqMonk (Babraham Institute), with a bin size of 1 kb. Data was visualized using R or GraphPad Prism.

Strain and Plasmid Construction

Whenever transforming strains that already contained one or more markers, transformants were first selected then patched using the antibiotic for the resistance to the incoming construct. Retention of the original markers/constructs was then confirmed by testing the resistance to the original markers. Where necessary, sequencing of genes or regions was conducted to verify the presence/absence of key point mutations.

Strain DMR109 was constructed by transforming 168CA with chromosomal DNA from HM31 (4) and selecting for tetracycline resistance.

Strain DMR179 was constructed by transforming DMR178 with chromosomal DNA from HM40 (4) (selecting for neomycin resistance). The presence of *soj(G12V)* was verified by DNA sequencing using primers oDMR194 and oDMR197.

DMR181 was constructed by transforming DMR178 with chromosomal DNA from HM38 (4) (selecting for neomycin resistance). The presence of *soj(K16A)* was verified by DNA sequencing using primers oDMR194 and oDMR197.

DMR184 and DMR185 were constructed by transforming DMR178 with chDNA from strains TK421 and TK422, respectively, selecting for neomycin resistance. The presence of both the *soj* and *spo0J* point mutations were confirmed by DNA sequencing. TK421 and TK422 were gifted by Tomas Kloosterman.

Strains DMR190, DMR191 and DMR192 were constructed by transforming DMR178, DMR179 and DMR181, respectively, with chromosomal DNA from TK110 (5) and selecting for tetracycline resistance. The presence of the *soj* point mutations was verified by DNA sequencing.

Strain DMR206 was constructed by transformation of DMR109 (selecting for neomycin resistance) with 3-way PCR using primers oDMR225 and oDMR227 (fragment 1) and 168CA template DNA; oDMR229 and oDMR231 (fragment 2) and pSHP1 (gifted by Stijn Peters) template DNA; and oDMR232 and oDMR233 (fragment 3) and 168CA template DNA.

Strain DMR208 was constructed by transformation of DMR109 (selecting for neomycin resistance) with 3-way PCR using primers oDMR225 and oDMR227 (fragment 1) and DMR179 template DNA; oDMR229 and oDMR231 (fragment 2) and pHSP1 template DNA; and oDMR232 and 233 (fragment 3) and DMR179 template DNA.

Strain DMR210 was constructed by transformation of DMR109 with 3-way PCR using primers: oDMR225 and oDMR227 (fragment 1) and DMR181 template DNA; oDMR229 and oDMR231 (fragment 2) and pHSP1 template DNA; and oDMR232 and oDMR233 (fragment 3) and DMR181 template DNA. Select with neomycin.

A 3-way PCR using oDMR245-246 (Fragment 1, 168CA template), oDMR247-248 (Fragment 2, p7Z6 template (6)), oDMR249-250 (Fragment 3, 168CA template) was transformed into 168CA to generate strain DMR236.

DMR256 and DMR258 were constructed by backcrossing HM240 (7) or HM625 (7), respectively, into 168CA. The transformants were selected for spectinomycin resistance and neomycin sensitivity. Retention of the double integration at *amyE* was verified by testing the activity of α-amylase on starch plates.

Strains DMR267 and DMR270 were constructed by transforming DMR256 and DMR258, respectively, with DNA from DMR119 and selecting for erythromycin resistance. Retention of the double integration at *amyE* was verified by testing the activity of α-amylase on starch plates and resistance to spectinomycin.

DMR308 and DMR310 were constructed by transforming DM256 and DMR258, respectively, with a PCR product using oDMR262 and oDMR263 and template DNA from DMR363, selecting for erythromycin resistance. Retention of the double integration at *amyE* was verified by testing the activity of α-amylase on starch plates and resistance to spectinomycin.

Strains DMR312 and DMR314 were constructed by transforming DMR308 and DMR310, respectively, with a PCR product generated using primers oDMR260-261 and HM531 template DNA (selecting for chloramphenicol resistance). The presence of the *dna(V323D)* mutation was confirmed by DNA sequencing.

Strain DMR316 was constructed transforming 168CA with DNA from JWV048 (8) and selecting for kanamycin resistance.

Strains DMR337, DMR339 and DMR341 were generated by transforming DMR206, DMR208 and DMR210, respectively, with pJG23 and selecting for chloramphenicol resistance.

Strains DMR346, DMR350 and DMR354 were constructed by transforming DMR267, DMR270 and DMR3369, respectively, with DNA from DMR316 (selecting for kanamycin resistance). Retention of the double integration at *amyE* was verified by testing the activity of α-amylase on starch plates and resistance to spectinomycin.

Strain DMR363 was generated by transforming 168CA (selecting for erythromycin resistance) with PCR product generated using primers oDMR262 and oDMR263 and template DNA from a strain harbouring an *smc-mNG* construct at the native locus (a gift from Maki Kawai).

DMR369 was generated by transforming DMR267 with a PCR product generated using primers oDMR260-261 and selecting for chloramphenicol resistance. The presence of *dnaA(V323D*) was verified by sequencing.

Strains DMR375 and DMR377 were constructed by transforming DMR317 and DMR318, respectively, with a PCR product generated using oDMR262-263 and DMR363 template DNA, selecting for erythromycin resistance.

DMR270 was transformed with a PCR product generated using primers oDMR260-261 and HM531 template DNA, selecting for chloramphenicol resistance, to generate strain DMR413. The presence of *dnaA(V323D)* was confirmed by sequencing. Retention of the double integration at *amyE* was verified by testing the activity of α-amylase on starch plates and resistance to spectinomycin

Strain DMR434 was generated by transforming DMR413 with DNA from HM908 and selecting for tetracycline resistance. The retention of *dnaA(V323D)* and the double integration at *amyE* were confirmed by DNA sequencing and testing the activity of α-amylase on starch plates, respectively.

Strains DMR447, DMR449 and DMR456 were generated by transforming DMR206, DMR208 and DMR210, respectively, with a PCR product generated using primers oDMR277 and oDMR233 and template DNA from strain 2640 (9), selecting for spectinomycin resistance then neomycin sensitivity.

DMR457, DMR459 and DMR461 were constructed by transforming DMR363, DMR312 and DMR314, respectively, with a PCR product generated using primers oDMR245 and oDMR250 and template DNA from strain TK110 (select for tetracycline resistance). The presence of the double integration at *amyE* was confirmed by testing the activity of α-amylase on starch plates.

DMR464, DMR466 and DMR468 were constructed by transforming DMR363, DMR312 and DMR314, respectively, with a PCR product generated using primers oDMR225 and oDMR233 and template DNA from strain HM748 (2) (select for neomycin resistance). The presence of the double integration at *amyE* was confirmed by testing the activity of α-amylase on starch plates.

Strains DMR473, DMR474 and DMR475 were constructed by transforming DMR369 with chDNA from HM748 (2), DMR317 and DMR318, respectively, and selecting for neomycin resistance. The presence of the point mutations in *dnaA* and *soj* were confirmed by DNA sequencing. The presence of the double integration at *amyE* was confirmed by testing the activity of α-amylase on starch plates.

Strains DMR476, DMR477 and DMR478 were generated by transforming DMR413 with chDNA from HM748 (2), DMR317 and DMR318, respectively, selecting for neomycin resistance. The presence of the point mutations in *dnaA* and *soj* were confirmed by DNA sequencing. The presence of the double integration at *amyE* was confirmed by testing the activity of α-amylase on starch plates.

Strain HM531 was constructed by transforming 168ED with DNA from HM528 (7) and selecting for chloramphenicol resistance. The presence of *dnaA(V323D*) was verified by DNA sequencing.

Strain HM908 was constructed by transforming 168CA with DNA from JWV044 (8) and selecting for tetracycline resistance.

TK230 was gifted to us by Tomas Kloosterman. The presence of the point mutation in *spo0J* was verified by sequencing.

Plasmid pJG23 was constructed by initially generating an empty vector (pJG95) though linearization of pAPNC213-cat (gifted by Clare Willis) with primers oJWG041 and oJWG007, and then the RBS

inserted by In-fusion cloning (Clontech) using annealed primers oJWG039-040. The mScarlet-I (10), amplified using oJWG001-002, was inserted into pJG95 after plasmid linearisation using oJWG005-006, generating pJG6. pJG23 was then completed by linearisation of pJG6 with oJWG051-052, while DivIVA was amplified from 168CA using oJWG071-072 and inserted by In-fusion cloning.

References

1. Schindelin J*, et al.* (2012) Fiji: an open-source platform for biological-image analysis. *Nat. Methods* 9(7):676-682.
2. Tinevez JY*, et al.* (2017) TrackMate: An open and extensible platform for single-particle tracking. *Methods* 115:80-90.
3. Vischer NO*, et al.* (2015) Cell age dependent concentration of *Escherichia coli* divisome proteins analyzed with ImageJ and ObjectJ. *Front Microbiol* 6:586.
4. Murray H & Errington J (2008) Dynamic control of the DNA replication initiation protein DnaA by Soj/ParA. *Cell* 135(1):74-84.
5. Kloosterman TG*, et al.* (2016) Complex polar machinery required for proper chromosome segregation in vegetative and sporulating cells of *Bacillus subtilis*. *Mol. Microbiol.* 101(2):333-350.
6. Yan X, Yu HJ, Hong Q, & Li SP (2008) Cre/lox system and PCR-based genome engineering in *Bacillus subtilis*. *Appl. Environ. Microbiol.* 74(17):5556-5562.
7. Scholefield G, Whiting R, Errington J, & Murray H (2011) Spo0J regulates the oligomeric state of Soj to trigger its switch from an activator to an inhibitor of DNA replication initiation. *Mol. Microbiol.* 79(4):1089-1100.
8. Veening JW, Murray H, & Errington J (2009) A mechanism for cell cycle regulation of sporulation initiation in *Bacillus subtilis*. *Genes Dev.* 23(16):1959-1970.
9. Autret S, Nair R, & Errington J (2001) Genetic analysis of the chromosome segregation protein Spo0J of *Bacillus subtilis*: evidence for separate domains involved in DNA binding and interactions with Soj protein. *Mol. Microbiol.* 41(3):743-755.
10. Bindels DS*, et al.* (2017) mScarlet: a bright monomeric red fluorescent protein for cellular imaging. *Nat. Methods* 14(1):53-56.

Fig. S1

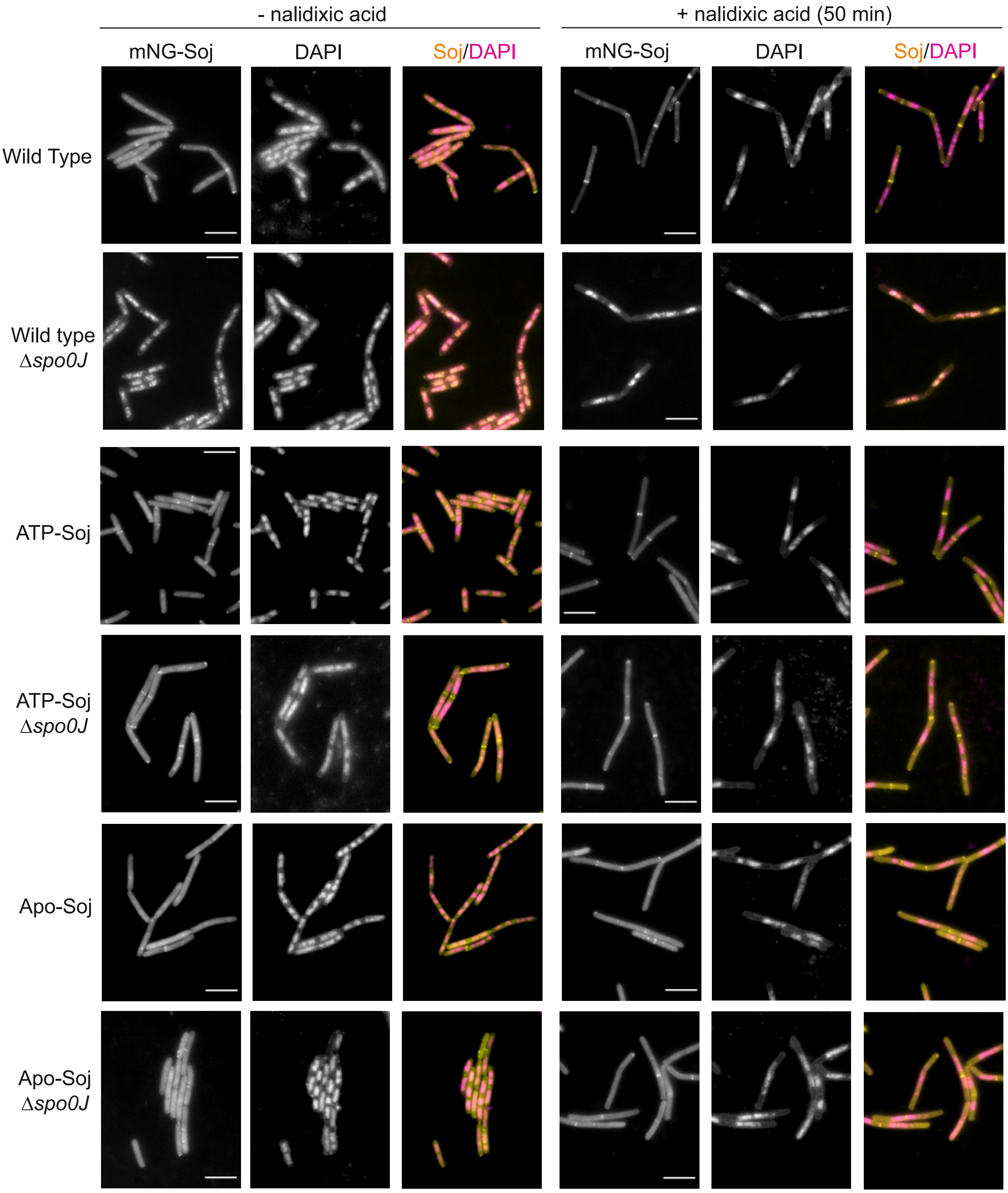

Fig. S1. Localisation of Soj in the presence and absence of *spo0J*. Representative images showing the localisation pattern of mNG-Soj +/- Δ*spo0J*. Nucleoids were visualised by staining with DAPI (1 μg/ml final). Nalidixic acid (10 μg/ml final) was added to growing cultures for 50 mins prior to imaging. Scale bar = 5 μm. Strains used: DMR206, DMR208, DMR210, DMR447, DMR449, DMR456.

Fig. S2

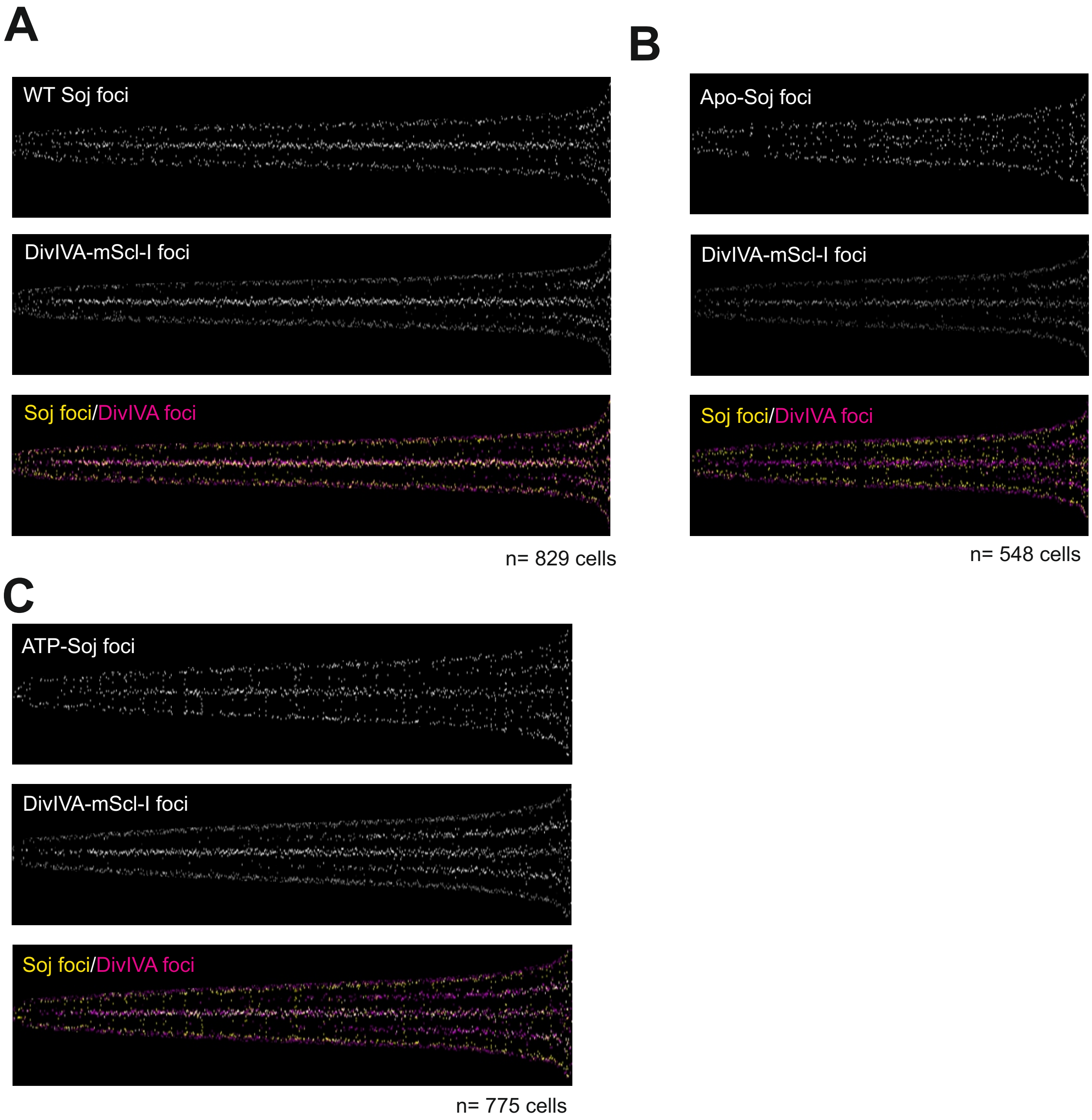

Fig. S2. Soj and DivIVA co-localisation. Fluorescent plots show the relative cellular positions of: (B) wild type mNG-Soj and DivIVA-mScl-I; (C) Apo-Soj and DivIVA-mScl-I; (D) ATP-Soj and DivIVA-mScl-I. In all cases, cells were sorted by shortest to longest. The DivIVA-mScl-I signal was used as a proxy for the cell boundaries/poles. Strains used: DMR337, DMR339. DMR341.

Fig. S3

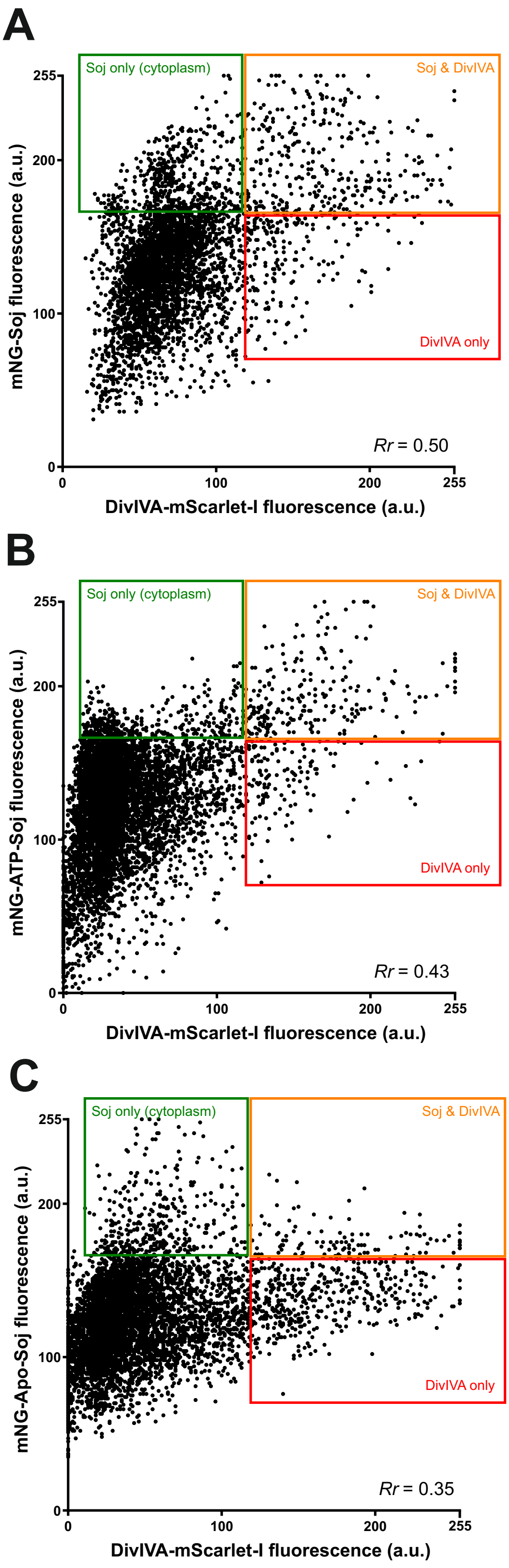

Fig. S3. Co-localisation analysis of mNG-Soj and DivIVA-mScl-I. The graphs show a fluorescence intensity correlation (pixel by pixel) of the Soj and DivIVA signal for: (A) wild type Soj and DivIVA; (B) ATP-Soj and DivIVA; (C) Apo-Soj and DivIVA. Images used are those from Fig. 2. The Pearson's correlation coefficient (*Rr*) is also shown. Green boxes = pixels corresponding to Soj only signal; orange boxes = pixels corresponding to co-localised Soj and DivIVA; red boxes = pixels corresponding to DivIVA only signal. Strains used: DMR337, DMR339, DMR341.

Fig. S4

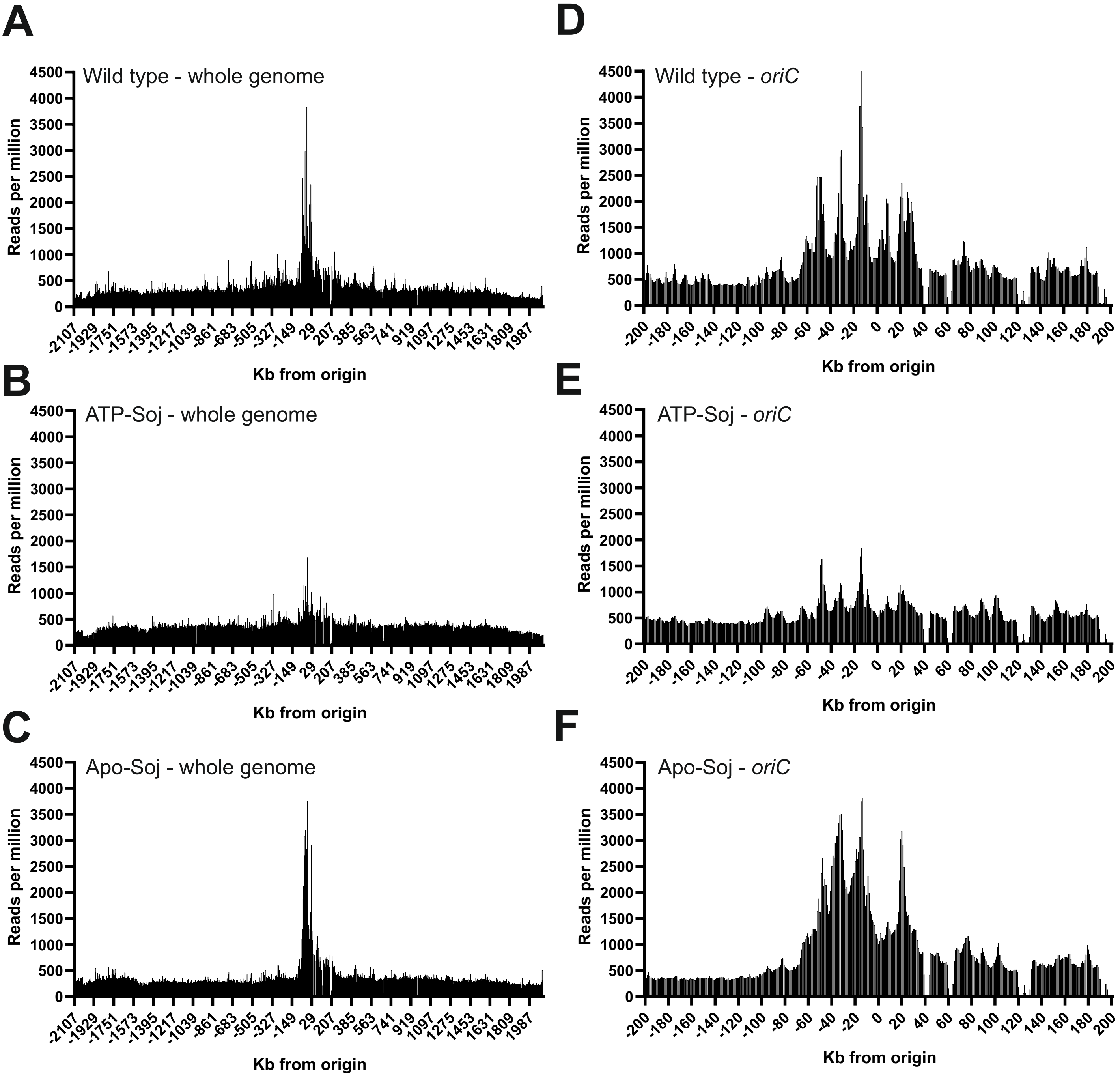

Fig. S4. ChIP-Seq reveals altered enrichment of SMC at *oriC* during sporulation. ChIP-Seq was conducted against ScpB 3 h after re-suspension in sporualtion salts. Whole genome plots are shown for (A) wild type; (B) ATP-Soj; (C) Apo-Soj. A close up of the origin region (+/- 200 Kb) is shown for (D) wild type; (E) ATP-Soj; (F) Apo-Soj. To lock the chromosome in its initial capture orientation, all strains also contained the *spoIIIE36* mutation. Strains used: DMR178, DMR179, DMR181.

Fig. S5

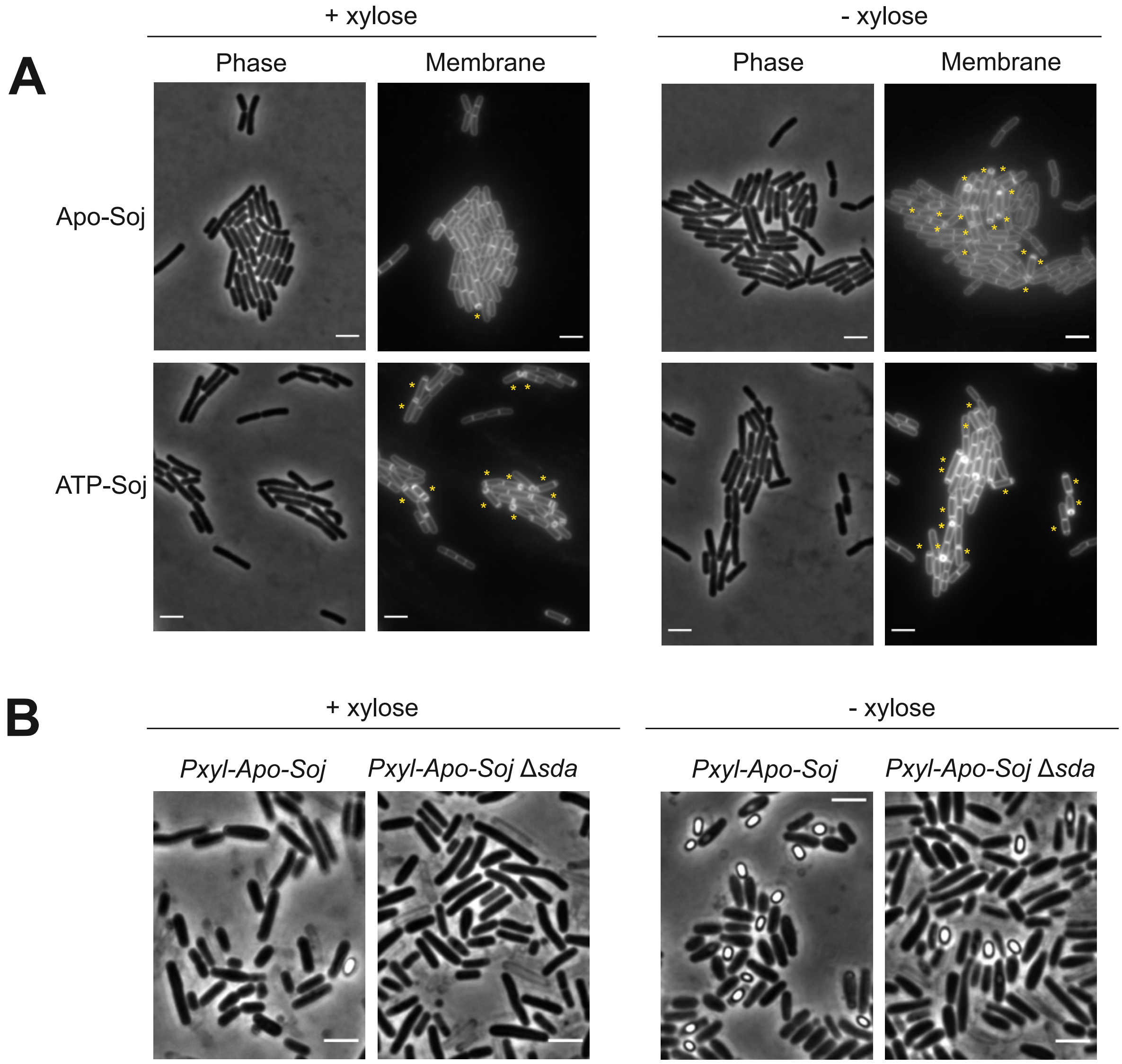

C

Time Point during sporulation	Asymmetric Division	Mean intensity of P_{spoIIA}-*mcherry*
ATP-Soj +xyl, 240min	Yes	684
ATP-Soj –xyl, 240min	Yes	2018
Apo-Soj +xyl, 240min	No	618
Apo-Soj –xyl, 240min	Yes	2005

Fig. S5. The Apo-Soj monomer is dominant negative over wild type. (A) Representative images showing cells in which Apo-Soj (top panels) and ATP-Soj (bottom panels) were either overexpressed (+ xylose) or not (- xylose) 100 mins after re-suspension in sporulation salts. For + xylose, 0.5 % xylose was added 15 mins prior to (and during) re-suspension in sporulation salts. Membranes were visualised using FM5-95. Yellow asterisks show examples of cells containing asymmetric septa. Scale bar = 3 µm. (B) Representative images of cells showing the dominant negative effect is not lost in Δ*sda*. + xylose = 0.5 % xylose in plates; - xylose = no induction. Scale bar = 3 µm. (C) Representative fluorescence intensities showing the induction of a P_{spoIIA}-*mcherry*. + xyl = addition of 0.5 % xylose to culture 15 mins prior to (and during) re-suspension in sporulation salts. - xyl = no induction. Strains used: DMR350, DMR354, DMR369, DMR413, DMR434.

Fig. S6

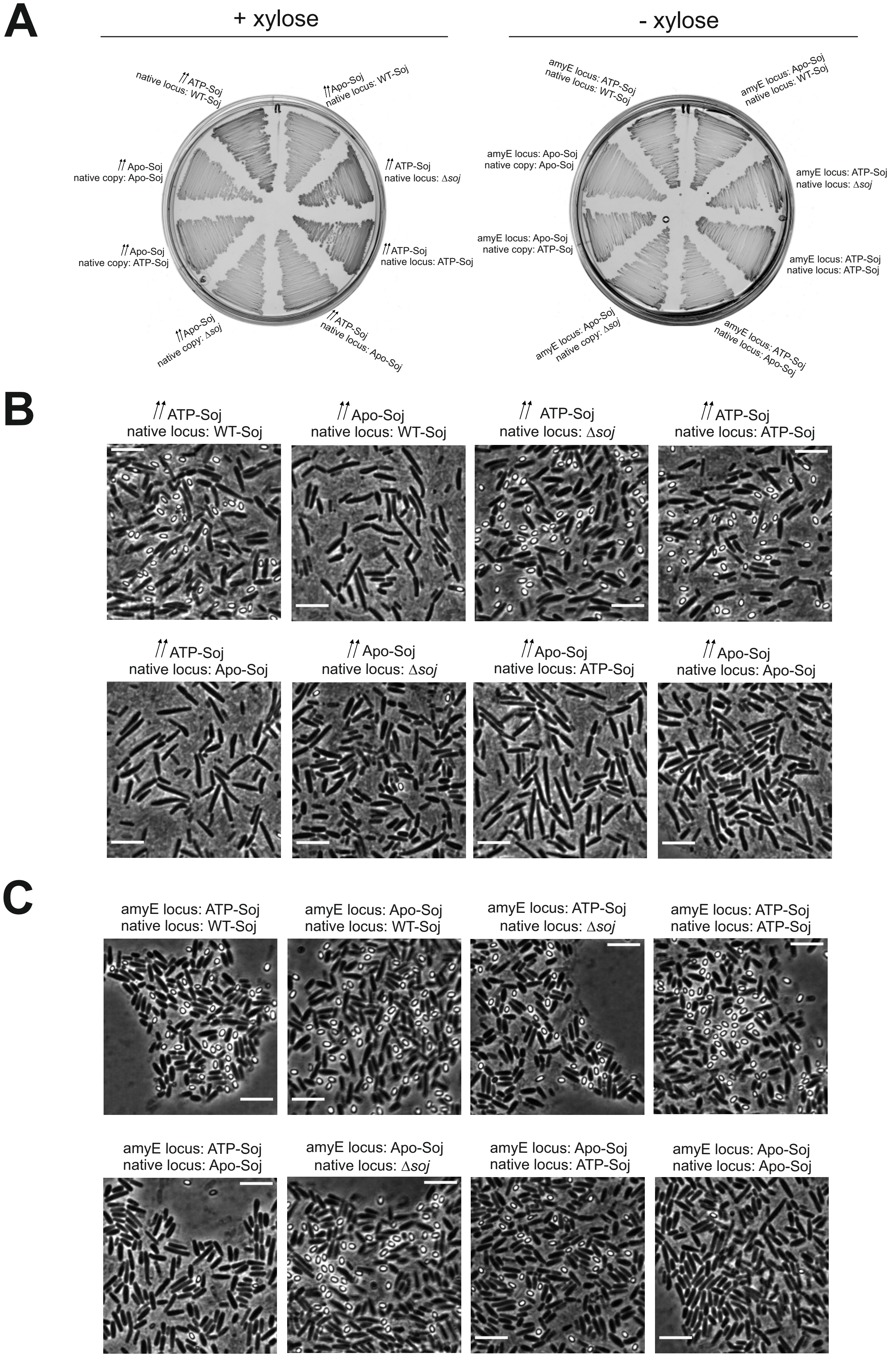

Fig. S6. Apo-Soj is dominant to wild type and ATP-Soj. (A) Nutrient agar plates +/- 0.5 % xylose to induce overexpression (indicated by double up arrows) of ATP-Soj or Apo-Soj at the non-essential amyE locus. The native locus contained either wild type, ATP-Soj, Apo-Soj or Δ*soj*. Phase images of cells from: (B) +xylose or (C) -xylose plates from A, showing the presence or absence of phase bright spores in each condition. Scale bar = 3 μm. Strains used: DMR369, DMR413, DMR473,DMR474, DMR475, DMR476, DMR477, DMR478.

Fig. S7

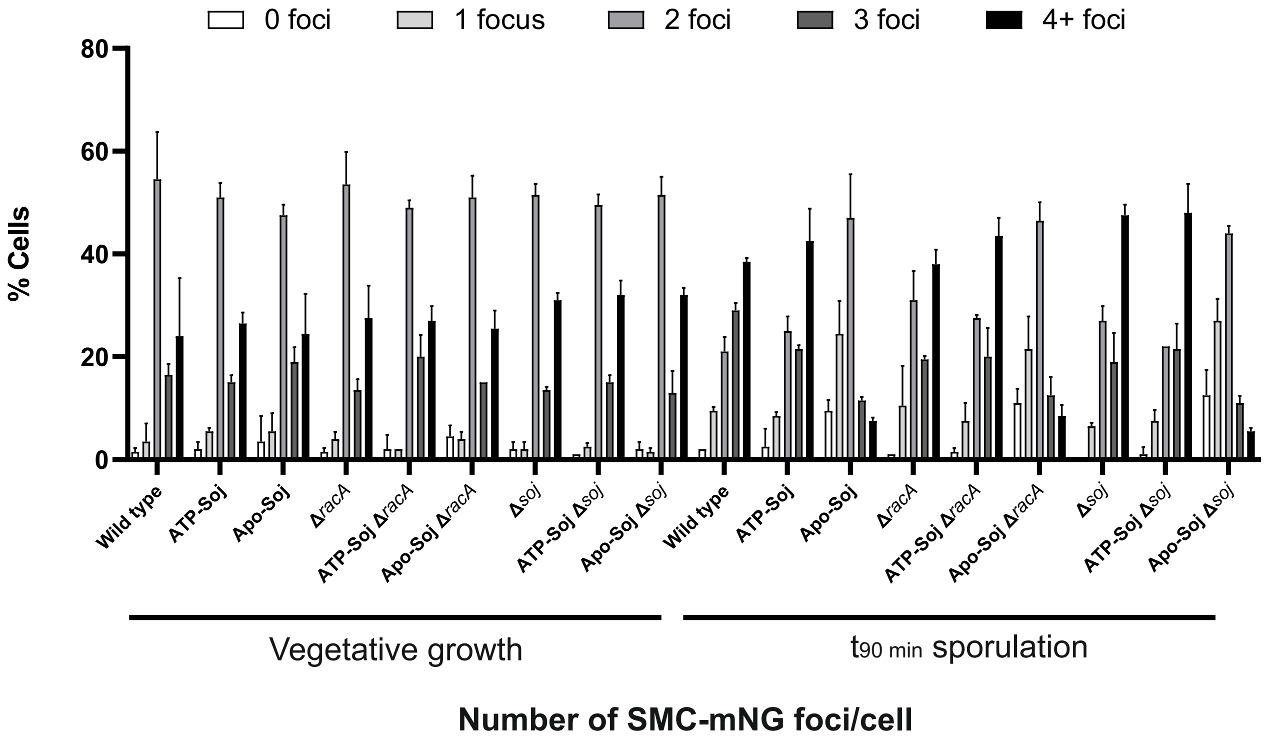

Fig. S7. SMC redistribution is not affected by deletion of native *racA* or *soj*. Bar chart showing the number of SMC-mNG foci detected per cell in each mutant at each time point. At least 100 cells were counted per repeat (n=2), with mean scores shown. Error bars show the standard deviations. Strains used: DMR312, DMR314, DMR363, DMR457, DMR459, DMR461, DMR464, DMR466, DMR468.

Fig. S8

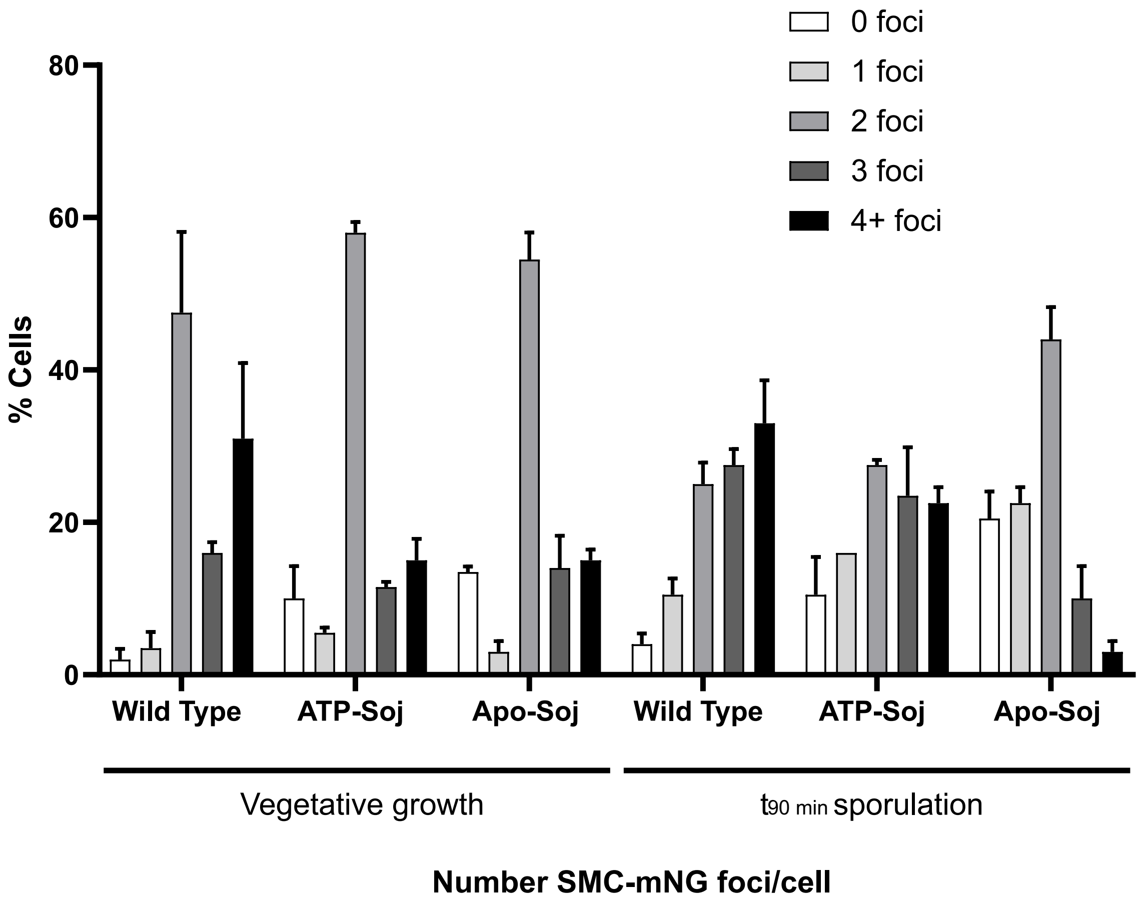

Fig. S8. SMC complex distribution is altered during sporulation. Bar chart showing the number of SMC-mNG foci per cell in wild type, ATP-Soj and Apo-Soj mutants expressed as single copies from their native locus. At least 100 cells were counted for each time point (n = 2) and average values are shown. Error bars show standard deviations. Strains used: DMR363. DMR375, DMR377.

Fig. S9

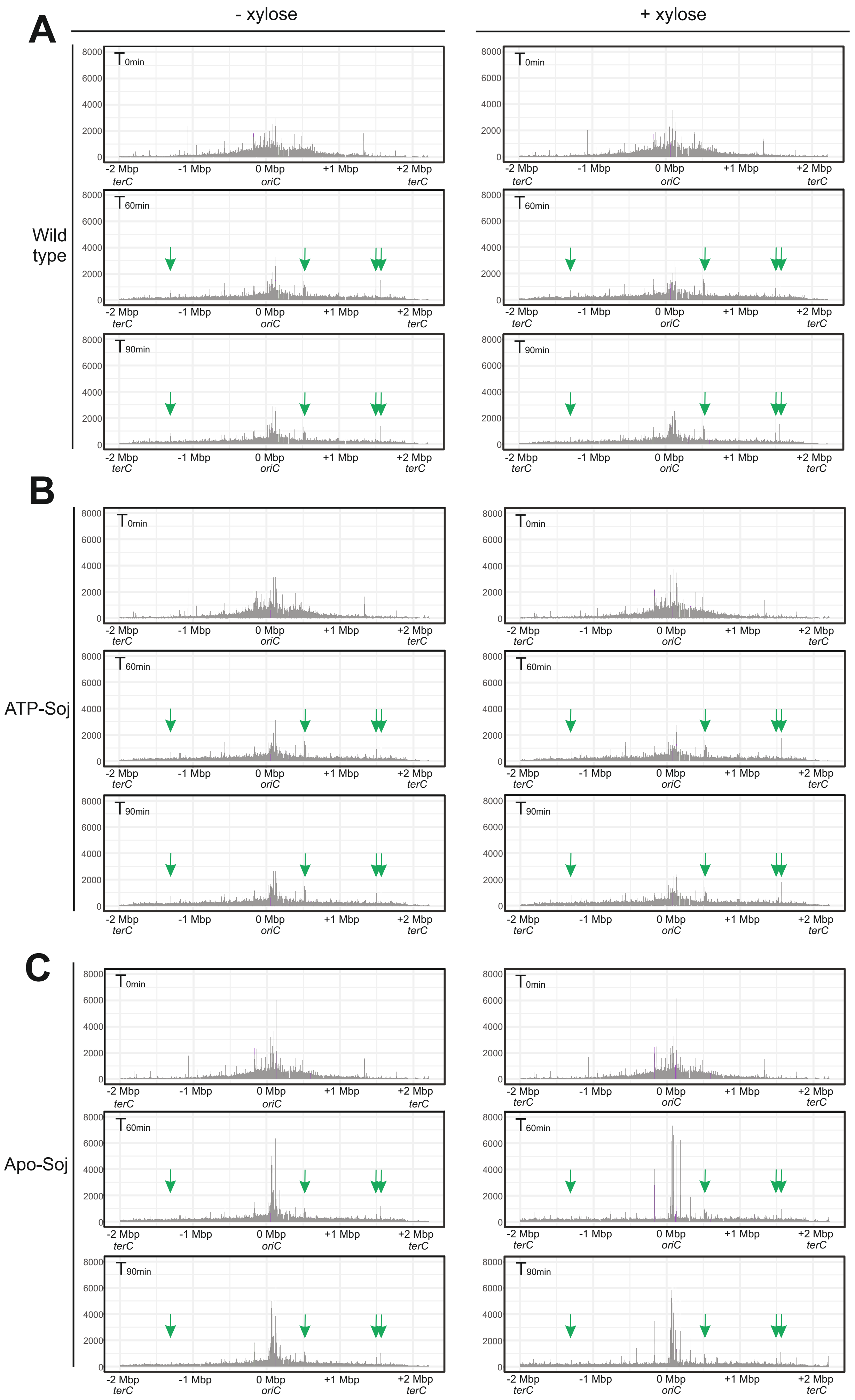
A
- xylose
+ xylose
Wild type
B
ATP-Soj
C
Apo-Soj
T0min
T60min
T90min
8000
6000
4000
2000
0
-2 Mbp
terC
-1 Mbp
0 Mbp
oriC
+1 Mbp
+2 Mbp
terC

Fig. S9. Whole genome anti-ScpB ChIP-Seq plots during early sporulation. Whole genome ChIP-Seq plots for (A) wild type; (B) ATP-Soj; (C) Apo-Soj. Cells were sporulated in the presence (+ xylose) or absence (- xylose) of 0.5 % xylose. T = minutes after re-suspension in sporulation salts. Green arrows = peaks specific to sporulation in all conditions. Purple lines = *parS* sites. Plots show reads per million. Strains used: DMR363, DMR312, DMR314.

Fig. S10

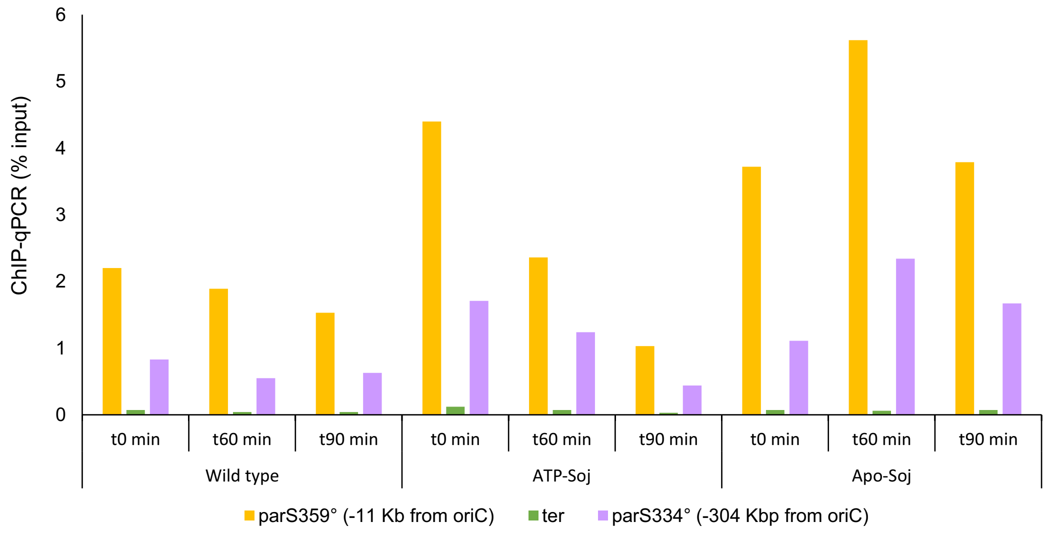

Fig. S10. ChIP-qPCR reveals enrichment of SMC at *parS* sites during sporulation in Apo-Soj. Graph showing α-scpB ChIP-qPCR in wild type (DMR363), ATP-Soj (DMR312) and Apo-Soj (DMR314), at three genomic locations: the *parS* site at 359° (*parS*359°; yellow bars), the terminus (ter; green bars), and the *parS* site at 334° (parS334°; purple bars). The time during sporulation the samples were taken is given in minutes.

Fig. S11

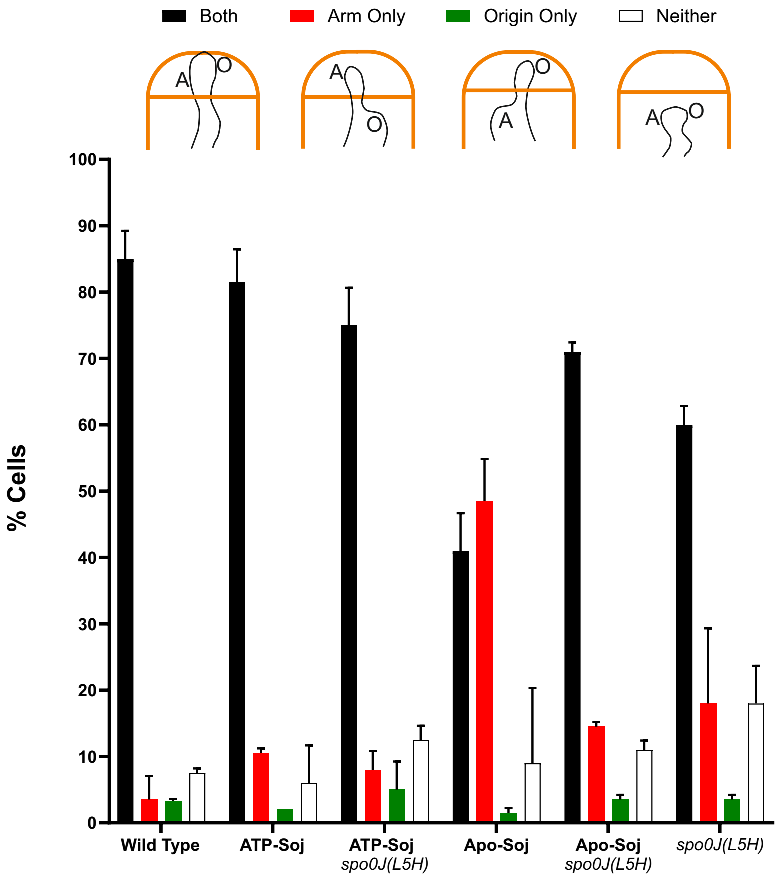

Fig. S11. Interaction between Soj and Spo0J is critical for Soj-mediatedchromosome trapping. Chromosome trapping assay to assess prespore localisation of armor origin localised fluorescent markers. n=2, at least 100 cells were counted per strain. Strains used: DMR178, DMR179, DMR181,DMR184, DMR185, TK230.

Table S1. Strains used in this study.

All strains are derivatives of 168CA, unless stated otherwise.

Strain	Genotype	Source
168CA	*trpC2*	(1)
168ED	*trpC2*	(2)
DMR109	*trpC2 Δ(soj spo0J)::tet*	This study.
DMR119	*trpC2 ΔracA::erm*	(3)
DMR178	*trpC2 spoIIIE36::cat yycR::(PspoIIQ-yfp (-7) spc) ywjI::(PspoIIQ-cfp (-35) erm)*	(3)
DMR179	*trpC2 (soj(G12V))::neo spoIIIE36::cat yycR::(PspoIIQ-yfp spc) ywjI::(PspoIIQ-cfp (-35) erm)*	This study.
DMR181	*trpC2 (soj(K16A))::neo spoIIE36::cat yycR::(PspoIIQ-yfp (-7) spc) ywjI::(PspoIIQ-cfp (-35) erm)*	This study.
DMR184	*trpC2 (soj(K16A) spo0J(L5H))::neo spoIIIE36::cat yycR::(PspoIIQ-yfp spc) ywjI::(PspoIIQ-cfp erm)*	This study.
DMR185	*trpC2 (soj(G12V) spo0J(L5H))::neo spoIIIE36::cat yycR::(PspoIIQ-yfp spc) ywjI::(PspoIIQ-cfp erm)*	This study.
DMR190	*trpC2 spoIIIE36::cat yycR::(PspoIIQ-yfp (-7) spc) ywjI::(PspoIIQ-cfp (-35) erm) ΔracA::tet*	This study.
DMR191	*trpC2 (soj(G12V))::neo spoIIIE36::cat yycR::(PspoIIQ-yfp spc) ywjI::(PspoIIQ-cfp (-35) erm) ΔracA::tet*	This study.
DMR192	*trpC2 (soj(K16A))::neo spoIIIE36::cat yycR::(PspoIIQ-yfp (-7) spc) ywjI::(PspoIIQ-cfp (-35) erm) ΔracA::tet*	This study.
DMR206	*trpC2 mNeonGreen-soj::neo*	This study.
DMR208	*trpC2 mNeonGreen-soj(G12V)::neo*	This study.
DMR210	*trpC2 mNeonGreen-soj(K16A)::neo*	This study.
DMR236	*ΔracA::zeo*	This study.
DMR256	*trpC2 amyE::(spc Pxyl-sojG12V)*	This study.
DMR258	*trpC2 amyE::(spc Pxyl-sojK16A)*	This study.
DMR267	*trpC2 amyE::(spc Pxyl-sojG12V) ΔracA::erm*	This study.
DMR270	*trpC2 amyE::(spc Pxyl-sojK16A) ΔracA::erm*	This study.
DMR308	*trpC2 smc-mNeonGreen::erm amyE::(spc Pxyl-sojG12V)*	This study.
DMR310	*trpC2 smc-mNeonGreen::erm amyE::(spc Pxyl-sojK16A)*	This study.
DMR312	*trpC2 smc-mNeonGreen::erm amyE::(spc Pxyl-sojG12V) dnaA(V323D)::cat*	This study.
DMR314	*trpC2 smc-mNeonGreen::erm amyE::(spc Pxyl-sojK16A) dnaA(V323D)::cat*	This study.
DMR316	*trpC2 sacA::(PspoIIA-mcherry kan)*	This study.
DMR317	*trpC2 soj(G12V)::neo*	(4)
DMR318	*trpC2 soj(K16A)::neo*	(4)
DMR337	*trpC2 mNG-soj::neo aprE::(Pspac-divIVA-mscarlet-I cat)*	This study

DMR339	*trpC2 mNG-soj(G12V)::neo aprE::(Pspac-divIVA-mscarlet-I cat)*	This study.
DMR341	*trpC2 mNG-soj(K16A)::neo aprE::(Pspac-divIVA-mscarlet-I cat)*	This study.
DMR346	*trpC2 ΔracA::erm amyE::(spc Pxyl-sojG12V) sacA::(PspoIIA-mcherry kan)*	This study.
DMR350	*trpC2 ΔracA::erm amyE::(spc Pxyl-sojK16A) sacA::(PspoIIA-mcherry kan)*	This study.
DMR354	*trpC2 ΔracA::erm amyE::(spc Pxyl-sojG12V) dnaA(V323D)::cat sacA::(PspoIIA-mcherry kan)*	This study.
DMR363	*trpC2 smc-mNeonGreen::erm*	This study.
DMR369	*trpC2 ΔracA::erm amyE::(spc Pxyl-sojG12V) dnaA(V323D)::cat*	This study.
DMR375	*trpC2 soj(G12V)::neo smc-mNeonGreen::erm*	This study.
DMR377	*trpC2 soj(K16A)::neo smc-mNeonGreen::erm*	This study.
DMR413	*trpC2 ΔracA::erm amyE::(spc Pxyl-sojK16A) dnaA(V323D)::cat*	This study.
DMR434	*trpC2 ΔracA::erm amyE::(spc Pxyl-sojK16A) dnaA(V323D)::cat Δsda::tet*	This study.
DMR447	*trpC2 (mNeonGreen-soj Δspo0J)::spc*	This study.
DMR449	*trpC2 (mNeonGreen-soj(G12V) Δspo0J)::spc*	This study.
DMR456	*trpC2 (mNeonGreen-soj(K16A) Δspo0J)::spc*	This study.
DMR457	*trpC2 smc-mNG::erm ΔracA::tet*	This study.
DMR459	*trpC2 smc-mNeonGreen::erm amyE::(spc Pxyl-sojG12V) dnaA(V323D)::cat ΔracA::tet*	This study.
DMR461	*trpC2 smc-mNeonGreen::erm amyE::(spc Pxyl-sojK16A) dnaA(V323D)::cat ΔracA::tet*	This study.
DMR464	*trpC2 smc-mNG::erm Δsoj::neo*	This study.
DMR466	*trpC2 smc-mNeonGreen::erm amyE::(spc Pxyl-sojG12V) dnaA(V323D)::cat Δsoj::neo*	This study.
DMR468	*trpC2 smc-mNeonGreen::erm amyE::(spc Pxyl-sojK16A) dnaA(V323D)::cat Δsoj::neo*	This study.
DMR473	*trpC2 ΔracA::erm amyE::(spc Pxyl-sojG12V) dnaA(V323D)::cat Δsoj::neo*	This study
DMR474	*trpC2 ΔracA::erm amyE::(spc Pxyl-sojG12V) dnaA(V323D)::cat soj(G12V)::neo*	This study.
DMR475	*trpC2 ΔracA::erm amyE::(spc Pxyl-sojG12V) dnaA(V323D)::cat soj(K16A)::neo*	This study.
DMR476	*trpC2 ΔracA::erm amyE::(spc Pxyl-sojK16A) dnaA(V323D)::cat Δsoj::neo*	This study.
DMR477	*trpC2 ΔracA::erm amyE::(spc Pxyl-sojK16A) dnaA(V323D)::cat soj(G12V)::neo*	This study.

DMR478	*trpC2 ΔracA::erm amyE::(spc Pxyl-sojK16A) dnaA(V323D)::cat soj(K16A)::neo*	This study.
HM531	*trpC2 dnaA(V323D)::cat* (168ED)	This study.
HM908	*trpC2 Δsda::tet*	This study.
TK110	*ΔracA::tet*	(3).
TK230	*spo0J(L5H) spoIIIE36::cat yycR::(PspoIIQ-yfp (-7) spc) ywjI::(PspoIIQ-cfp (-35) erm)*	This study.
TK421	*trpC2 (soj(K16A) spo0J(L5H))::neo*	This study.
TK422	*trpC2 (soj(G12V) spo0J(L5H))::neo*	This study.

1. Kunst F, *et al.* (1997) The complete genome sequence of the gram-positive bacterium Bacillus subtilis. *Nature* 390(6657):249-256.
2. Kobayashi K, *et al.* (2003) Essential *Bacillus subtilis* genes. *Proc Natl Acad Sci U S A* 100(8):4678-4683.
3. Kloosterman TG, *et al.* (2016) Complex polar machinery required for proper chromosome segregation in vegetative and sporulating cells of *Bacillus subtilis*. *Mol. Microbiol.* 101(2):333-350.
4. Murray H & Errington J (2008) Dynamic control of the DNA replication initiation protein DnaA by Soj/ParA. *Cell* 135(1):74-84.

Table S2. Plasmids used in this study.

MCS = multi-cloning site. Descriptions denote the genotype upon transformation into *B. subtilis*.

Plasmid	Description	Source
p7Z6	*zeo cat*	(1)
pJG23	*aprE::(Pspac-divIVA-mScarlet cat)*	This study

1. Yan X, Yu HJ, Hong Q, & Li SP (2008) Cre/lox system and PCR-based genome engineering in *Bacillus subtilis*. *Appl. Environ. Microbiol.* 74(17):5556-5562.

Table S3. Oligonucleotides used in this study.

Primer	Sequence (5' → 3')
oDMR194	TCAATTATTTCCCTTCTGATTCGG
oDMR195	TTCAGCTTGCAGGGGCTGAGATCG
oDMR196	GAAGGTGTATTGCTGACAATGC
oDMR197	AACCCGTTGCAAAGGCTCACTGGGC
oDMR225	CCGCTTCTGTGGCGTTAATCG
oDMR227	CATATTATCCTCCTCTCCTTTCGAAACCATGATGTCACCTACTTTCACATG
oDMR229	GTACATGTTCATGTGAAAGTAGGTGACATCATGGTTTCGAAAGGAGAGGAG
oDMR231	GGTTCGTAATTGCTATGATTTTTCCCACCGTGGATCCTGAGCCGCTTCCTGA
oDMR232	TCAGGAAGCGGCTCAGGATCCACGGTGGGAAAAATCATAGCAATTAC
oDMR233	CTTGATCATGATCGAGGCAATGG
oDMR245	GTGCGGATACGTTCAGAGCGGTCACCAG
oDMR246	GGGGATCCACTAGTTCTAGAGCGGCCGGCGGTTCCCTTCCCGTTAGAC
oDMR247	CTTGTTTTGTCTAACGGGAAGGGAACCGCCGGCCGCTCTAGAACTAGTGGAT CC
oDMR248	CTTTAGGTTTGAAATTTGAACAGTGACAGCTCGAATTCTCAGTCCTGCTCCTC GGCC
oDMR249	CGTGGCCGAGGAGCAGGACTGAGAATTCGAGCTGTCACTGTTCAAATTTCAA ACC
oDMR250	CAAGCGGTACTTGATGCAATGTGGACACC
oDMR253	GACTTATTACAGATATCACACCGCC
oDMR260	CTTTGCTGCGTTTACGGTTATTCGGTTGG
oDMR261	CTACAAGCTGATCGGTCAGAACATCG
oDMR262	CGGCTATGAGTGAAGGAGATTTGACG
oDMR263	ATGACGTCTTGGCCTACAGC
oDMR277	GGGGCATGTTTGGCTTACATAG
oDMR279	TAATGTGTACGAATGGTAAGC
oDMR280	GGCTGTTCAATAGTGACG
oDMR281	GGCTGGAGTCCAGATACTTG
oDMR282	CGCGAATCAAATCGACAGC
oJWG001	gcgagctcgaattcgggctcaggaagcggctcaggatctATGGTCTCCAAAGGAGAGGC
oJWG002	gcttgggctaacgccTTATTTATACAGCTCATCCATACCACCCG

oJWG005	ggcgttagcccaagcgcatc
oJWG006	cgaattcgagctcgcccggg
oJWG007	ggatccccgggcgagctcg
oJWG039	tgacctgcaggcatgtagaaaggagattcctaggatgggatccccgggcgag
oJWG040	ctcgcccggggatcccatcctaggaatctcctttctacatgcctgcaggtca
oJWG041	catgcctgcaggtcaattgtgagcg
oJWG051	ggctcaggaagcggctcagg
oJWG052	cctaggaatctcctttctacatgcctgcag
oJWG071	aaggagattcctaggATGCCATTAACGCCAAATGATATTCACAAC
oJWG072	gccgcttcctgagccTTCCTTTTCCTCAAATACAGCGTCGACTTC
